# Supplementary material for: Fabricating strong and tough aramid fibers by small addition of carbon nanotubes
Source: Nat Commun. 2023 May 25;14:3019. doi: 10.1038/s41467-023-38701-4 (PMC10212957; doi:10.1038/s41467-023-38701-4)
Supplement: Supplementary file 1 — Supplementary Information [file 41467_2023_38701_MOESM1_ESM.pdf]

Supplementary Information

Fabricating Strong and Tough Aramid Fibers by Small Addition of Carbon Nanotubes

Jiajun Luo, Yeye Wen, Xiangzheng Jia, Xudong Lei, Zhenfei Gao, Muqiang Jian, Zhihua Xiao, Lanying, Li, Jiangwei Zhang, Tao Li, Hongliang Dong, Xianqian Wu*, Enlai Gao*, Kun Jiao*, Jin Zhang*

Correspondence to: wuxianqian@imech.ac.cn, enlaigao@whu.edu.cn, jiaokun-cnc@pku.edu.cn, jinzhang@pku.edu.cn

This PDF file includes:

Supplementary Methods

Supplementary Notes 1 to 4

Supplementary Figures 1 to 35

Supplementary Tables 1 to 15

Supplementary References

Supplementary Methods

Focused ion beam and scanning electron microscope tomography

Focused ion beam and scanning electron microscope tomography (FIB-SEMT) is an important approach for the study on the spatial distribution of structure[1]. The sample is repeatedly sliced with the FIB and each new cross-section is imaged with the SEM. Different from TEM tomography, high resolution and large volume size can be satisfied by FIB-SEMT at the same time. The quality of SEM imaging, the accuracy of FIB processing, and the stability of the system are the key factors for the excellent final result. The FIB-SEM dataset was collected using the Crossbeam 550 (ZEISS). The FIB was operated at 300 pA during the collection of the dataset. To ensure the accurate slice thickness of 5 nm, drift correction was automatically performed before each step. The excellent SEM images of the new cross-section were collected with the EHT 1.5 kV, and the SE2 detector with the grid was set to 350 V. Dwell time of 50 ns with a line average of 20 was performed. The pixel size was 4 nm. The pixel dimensions of the image were 2038×1536 pixels. The image stacking and three-dimensional (3D) reconstruction were performed with the professional software Dragonfly (ORS). The automatic structure segmentation, based on the boundary conditions, was performed using the deep learning module trained with U-Net model.

Wide angle X-ray scattering measurements

Wide angle X-ray scattering (WAXS) measurements were performed on a Xenocs Xeuss SAXS/WAXS system using an incident Cu-Kα X-ray beam perpendicular to the fiber axis. The wavelength dimension of the X-ray was 0.154 nm and the distance between the detector and the sample was 60 mm. The resulting analysis of the WAXS pattern was performed by Foxtrot software. The orientation degree is an important indicator for evaluating the alignment of fibers, which is calculated by the ordered crystals of certain crystal planes inside the fibers[2-5]. The orientation degree of crystalline orientation is calculated by integrating the curve against the azimuthal degree of WAXS patterns. One of the two peaks was then fitted with a Lorenz-peak function using Origin 2020b. Then, the full width at half maximum (FWHM) can be obtained from the fitted curves. The following equation was used to calculate the degree of orientation,

$$S = \frac{180 - \mathrm{FWHM}}{180}. \quad (1)$$

Small angle X-ray scattering measurements

Small angle X-ray scattering (SAXS) measurements were performed on a beam line (BL19U2) at Shanghai Synchrotron Radiation Facility. The wavelength dimension of the X-ray was 0.103 nm and the distance between the detector and the sample was 5800 mm. The resulting analysis of the SAXS patterns was performed by FIT two-dimensional (2D) software and xPolar software. SAXS can be used to study the scattering phenomenon in the small angle range and analyze the internal structure of fibers within the micro-size, such as the misorientation angle[6]. The angle between the microfiber and the fiber axis direction is defined as the misorientation angle (B_φ). B_φ was calculated from the SAXS equatorial streak feature with the Ruland streak method using equation:

$$B_{\text{obs}} = \frac{1}{s l_{\text{f}}} + B_{\varphi}, \tag{2}$$

where s is the scattering vector, B_{obs} is the full width at the half-maximum of the azimuthal profile, l_{f} is the fibril length, and s can be determined by equation:

$$s = \frac{2\sin\theta}{\lambda}, \tag{3}$$

where θ is the half value of the scattering angle, and λ is the wavelength dimension of the X-ray. All azimuthal distributions can be modeled by Lorentz functions.

Quasi-static mechanical property tests of monofilament

Quasi-static tensile tests were conducted on a Nano-Tensile Tester-testing system (SHIMADZU EZ-LX 5N) at a loading rate of 1 mm min^{-1}. Samples for tensile test were prepared by sticking fibers to rectangular paper frames with a gauge length of 20 mm using epoxy resin glue. The samples were loaded between the two clamp stages with the top clamp stage applying uniaxial tension on the samples along the vertical direction. The diameter of each specimen was measured by optical microscope before the tensile test. After the tensile test measurement, quantitative analysis of the modulus and toughness was carried out by Origin 2020b software. The modulus was equal to the slope of the curves at 0.5-0.75% strain, and the toughness was calculated by integrating the area of tensile curves. The values of tensile strength, modulus, elongation at break, and toughness are the average of 10 valid test results, where the few samples that broke near the clamps were excluded from the calculations.

Linear density tests of fiber yarns

The linear density of yarns was measured by the weighing method, which was calculated by equation:

$$D = W/L, \tag{4}$$

where D (tex), W (g), and L (km) are the linear density, the weight, and the length of yarns, respectively. The weight of dry yarns with a length of 1050 cm was measured by a precise analytical balance.

Mechanical tests of fiber yarns

Mechanical tests of fiber yarns were conducted on a Tester-testing system [CMT6103, MTS SYSTEMS (CHINA) CO., LTD] at a loading rate of 25 mm min^{-1}. The samples were loaded between the two clamp stages with a gauge length of 170 mm. The values of specific tensile strength (cN $dtex^{-1}$), modulus (cN $dtex^{-1}$), and elongation at break (%) are derived from 10 valid tests.

Stress relaxation measurements

The stress relaxation experiment of fibers was carried out based on the Agilent T150UTM nanotensile testing platform designed and produced by Agilent. The test sample was fixed on paper with a diamond hole in the middle with an epoxy resin glue, and the test length of the sample was 20 mm. The initial preload was set to 750 μN to ensure that the fiber was in a tight state during tension. The initial strain was set as 1.5%, and the initial loading was carried out at a loading strain rate of 1×10^{-3} s^{-1}, and the strain was maintained for 5000 s to fully relax. The tensile speed in the loading process was controlled by strain rate. Finally, we discuss the experimental results in terms of the curve of the normalized stress versus the relaxation time.

Other characterizations

The dispersion and detailed structure of SWNTs were investigated by SEM (FEI Quattro S, acceleration voltage 5–10 kV), TEM (FEI Tecnai F20; acceleration voltage 200 kV), Raman spectroscopy (Horiba, LabRAM HR 800, 532 nm laser wavelength), X-ray photoelectron spectroscopy (XPS; Kratos Analytical Axis-Ultra spectrometer with Al Kα X-ray source). The dispersion of SWNTs was investigated by Laser Particle Size Analyzer (BLUEWAVE S3500) and zeta potential instrument (Anton Paar, Austria). The radial cross-section of HAFs and

sa-SWNT-HAFs was cut by Ultramicrotome (Leica EM UC6). The axial cross-section of HAFs and sa-SWNT-HAFs was cut by FIB (ZEISS Crossbeam 550). The observation of SWNTs inside fibers was operated by TEM (FEI Tecnai F20; acceleration voltage 200 kV) and aberration-corrected atomic-resolved TEM (Titan Cubed Themis G2 300; 80 kV). The molecular weight of different polymer solutions was investigated by gel permeation chromatography (1260 Infinity II GPC/SEC) with columns in series (PL1110-6504). The thermal stability is studied by thermogravimetric analysis (TGA, STA 449 F5, Netzsh, Selb, Germany). The heating rate is 10°C min^{-1}, and the curves are recorded from room temperature to 900°C (the shielding gas and purge gas are nitrogen).

Supplementary Note 1: Further discussion on dispersion of SWNTs prepared from different process

Laser particle size analyzer and zeta potential instrument were usually used to characterize the dispersion of materials. D90 is used to express the corresponding particle size when the cumulative particle size distribution number of a sample reached 90% (**Supplementary Fig. 4a**). The zeta potential is used to express the stability of the material in solution (**Supplementary Fig. 4b**). The solution is generally considered well dispersed when its absolute value exceeds 30 mV. Due to the bundle morphology of SWNTs, the D90 of raw SWNTs is the highest, and the absolute value of zeta potential is the lowest, indicating that the raw SWNTs have the poorest dispersion. With the stepwise modification of SWNTs, the D90 gradually decreases, and the absolute values of zeta potential gradually increase, which means that the dispersion is gradually stable. Especially, the sc-SWNTs have the best dispersity. Although after the amination process of sc-SWNTs, the resultant sa-SWNTs can still maintain good dispersity.

Supplementary Note 2: Further discussion on XPS characterization of SWNTs

XPS spectra (**Fig. 2e**) demonstrated the successful preparation of sa-SWNT because of the presence of N *1s* peak in the curve of sa-SWNT. Additionally, due to the process of reoxidation of short-SWNTs, sc-SWNTs possess more reactive carboxyl groups by means of XPS-peak-differentation-imitating analysis of C *1s*, compared with long-SWNTs and short-SWNTs (**Supplementary Fig. 3a-d**). Therefore, after the amination reaction with ethylenediamine, sa-SWNTs have more nitrogen content compared with al-SWNTs and as-SWNTs (**Supplementary Fig. 3e, f**). Most importantly, the contents of nitrogen element in amide groups and amino groups are close to the same by means of XPS-peak-differentation-imitating analysis of N *1s*, which indicates that each ethylenediamine molecule tends to react with one carboxyl group of sc-SWNT and generates a free reactive amino group, rather than crosslinking two sc-SWNTs (**Fig. 3f**).

Supplementary Note 3: Further discussion on the addition and residual problems of PVP

In order to achieve better dispersion of SWNTs, PVP as a dispersant was added. Although SWNTs after modifications can be uniformly dispersed in water, they will agglomerate in the subsequent liquid nitrogen freeze-drying process, which results in the challenge to disperse them uniformly in DMAc. To avoid agglomeration of SWNTs during the freeze-drying process and achieve uniform dispersion of SWNTs in DMAc, PVP was added after the modification of SWNTs in water. In addition, because PVP exhibits good water solubility and low thermal decomposition temperature, it can be removed during the spinning process that includes two coagulation procedures (m_{water}:m_{DMAc} = 1:1 for primary coagulation bath and m_{water}:m_{DMAc} = 4:1 for secondary coagulation bath) after a water washing procedure in about half an hour and a heat treatment procedure (410°C). To provide quantitative experimental evidence, we did thermogravimetric analyses of PVP, HAFs and 1 wt% PVP-HAFs (**Supplementary Fig. 11**). To be specific, heterocyclic aramid spinning dopes with and without 1 wt% PVP were prepared and spun into fibers. The thermogravimetric analysis of PVP shows a significant weight loss when the temperature ranges from 350°C to 470°C, indicating that PVP can be removed in the heat treatment procedure (410°C). However, during the same temperature range, the thermogravimetric curves of HAFs and 1 wt% PVP-HAFs are almost identical, implying that there is almost no PVP residue in 1 wt% PVP-HAFs. On the other hand, even if there is a trace amount of PVP residue in HAFs, SWNTs don't prefer to bind with PVP compared to aramid chains, since our calculations show that the binding energy between the sp^2 carbon sheet and the heterocyclic aramid chain (46.2 meV atom^{-1}) is much higher than that between the sp^2 carbon sheet and the PVP (17.5 meV atom^{-1}).

Supplementary Note 4: Further discussion on porosities of HAFs and sa-SWNT-HAFs derived from FIB and FIB-SEMT

The void microstructure and porosities of HAFs and sa-SWNT-HAFs were characterized using FIB and FIB-SEMT. Due to the weak interaction between heterocyclic aramid chains, more voids are generated in HAFs during the process of wet spinning (**Fig. 3d, e, Supplementary Figs. 14a-c**). By contrast, with the addition of sa-SWNT, the sa-SWNT-HAFs possess fewer voids and keep a dense microstructure. In order to study the void distribution in detail, the 3D void microstructures of HAFs and sa-SWNT-HAFs were reconstructed using FIB-SEMT (**Supplementary Movies 1 to 4**). The results show that sa-SWNT-HAFs have a uniform void distribution and a lower porosity (0.0068%) than HAFs (0.017%) (**Supplementary Fig. 14d**). The compact structure obtained from wet-spinning is benefited from the strong interfacial interaction between heterocyclic aramid chains and sa-SWNTs, and the high degree of orientation and crystallinity induced by sa-SWNTs.

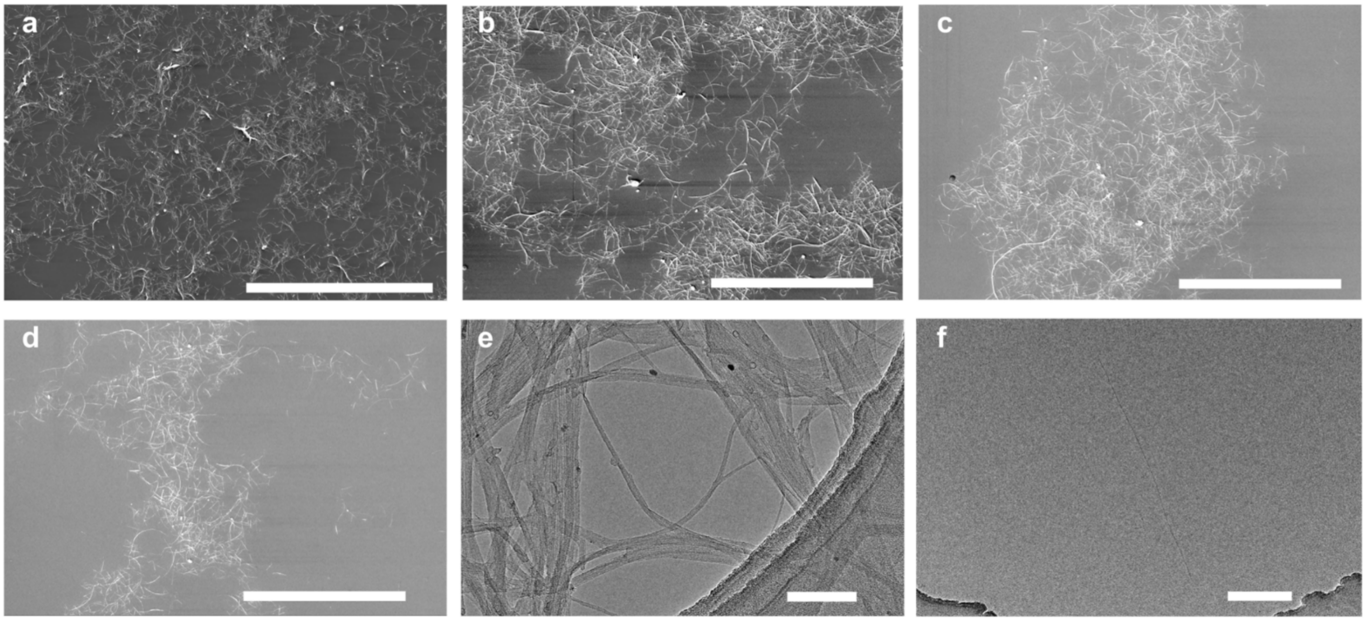

Supplementary Fig. 1 | Morphology characterization of SWNTs. SEM images of (**a**) raw SWNTs, (**b**) long-SWNTs, (**c**) short-SWNTs, and (**d**) sc-SWNTs. TEM images of SWNTs for (**e**) long-SWNTs and (**f**) sa-SWNTs. Scale bars, 50 μm in **a**; 10 μm in **b, c, d**; 100 nm in **e**; 200 nm in **f**.

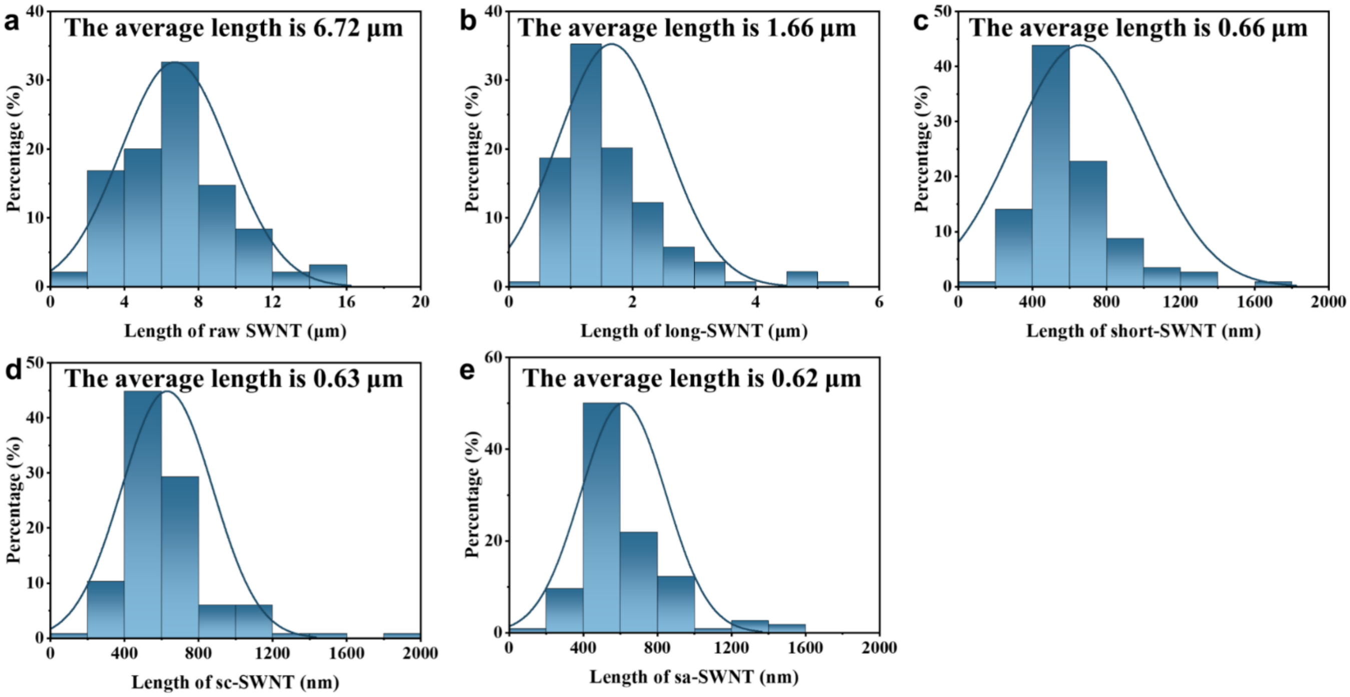

Supplementary Fig. 2 | Length distribution of SWNTs. The length distribution of (**a**) raw SWNTs, (**b**) long-SWNTs, (**c**) short-SWNTs, (**d**) sc-SWNTs, and (**e**) sa-SWNTs.

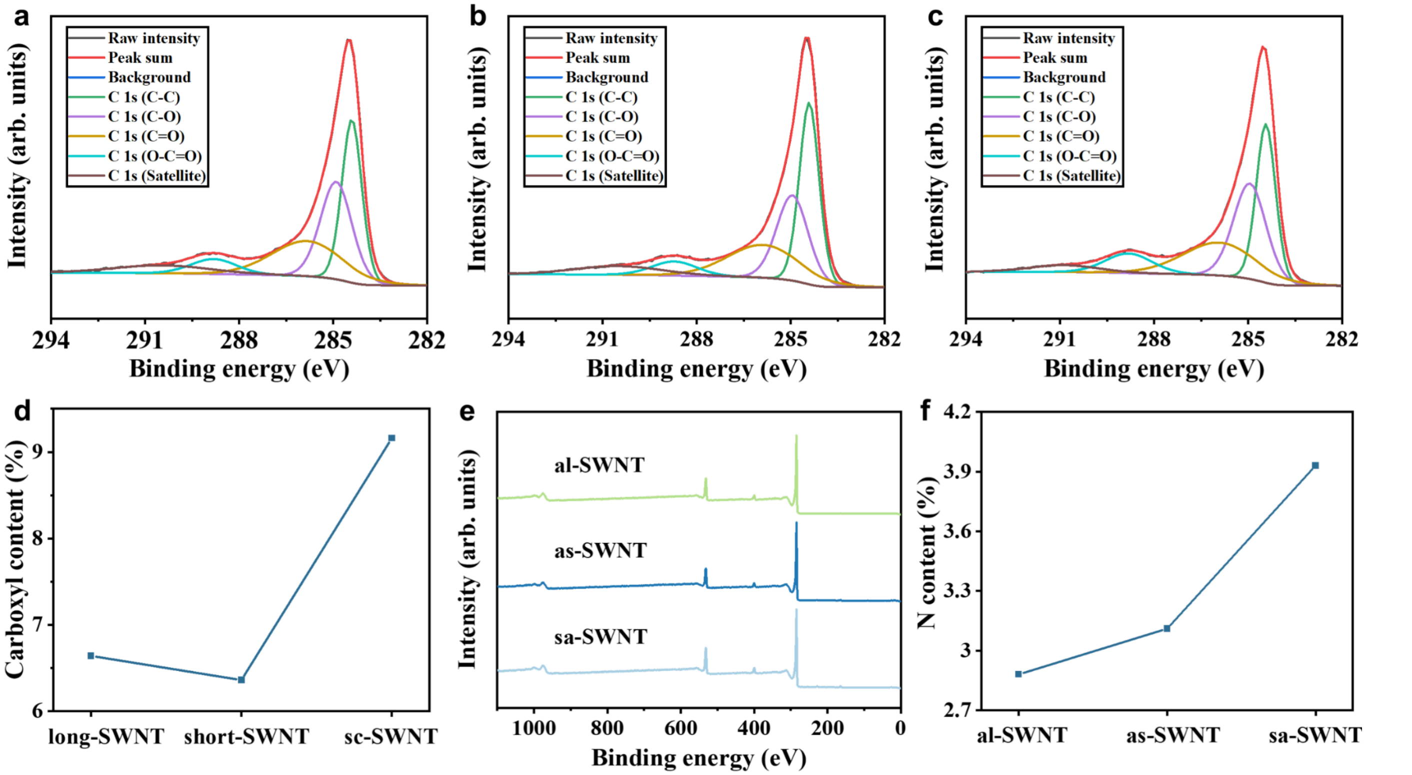

Supplementary Fig. 3 | Structure characterization of different SWNTs. XPS spectra of C *1s* of (**a**) long-SWNTs, (**b**) short-SWNTs, and (**c**) sc-SWNTs. **d,** Comparison of the carboxyl content of long-SWNTs, short-SWNTs, and sc-SWNTs. **e,** XPS spectra of different animated SWNTs. **f,** Comparison of the N content of different animated SWNTs.

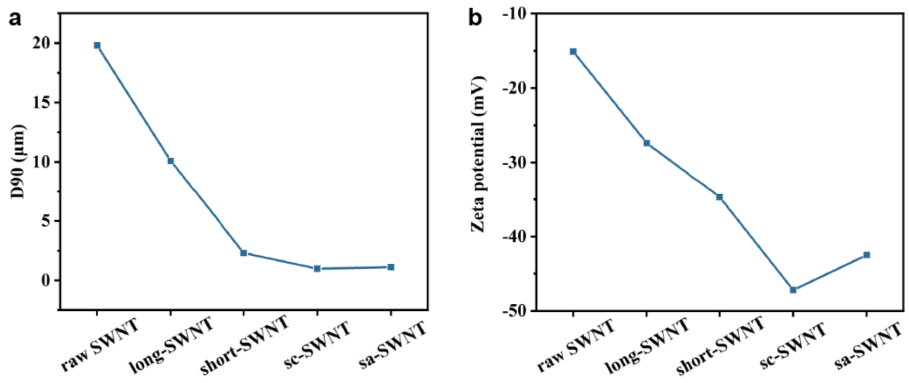

Supplementary Fig. 4 | Dispersion characterization of SWNTs prepared from different processes. a, D90 distribution of different SWNTs. **b,** Zeta potential of different SWNTs.

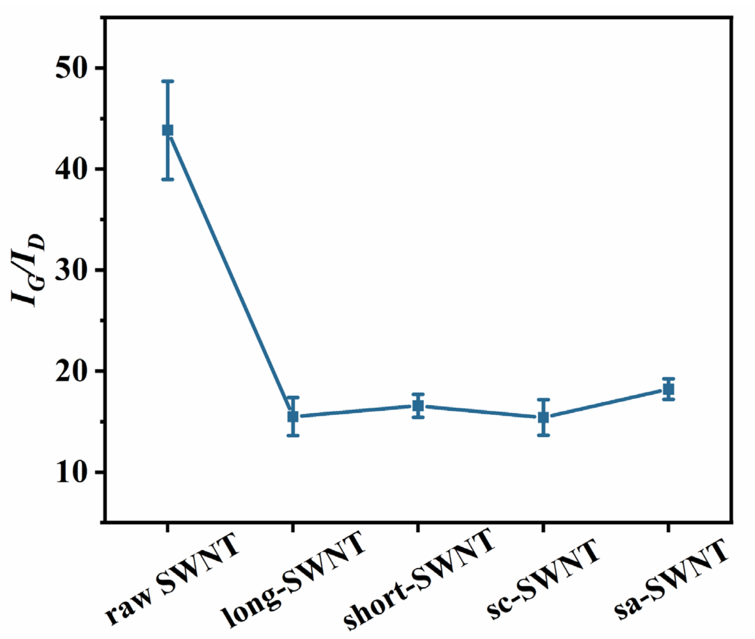

Supplementary Fig. 5 | Comparison of the I_G/I_D values of different SWNTs from Raman spectra analysis. Error bars indicate the standard deviation.

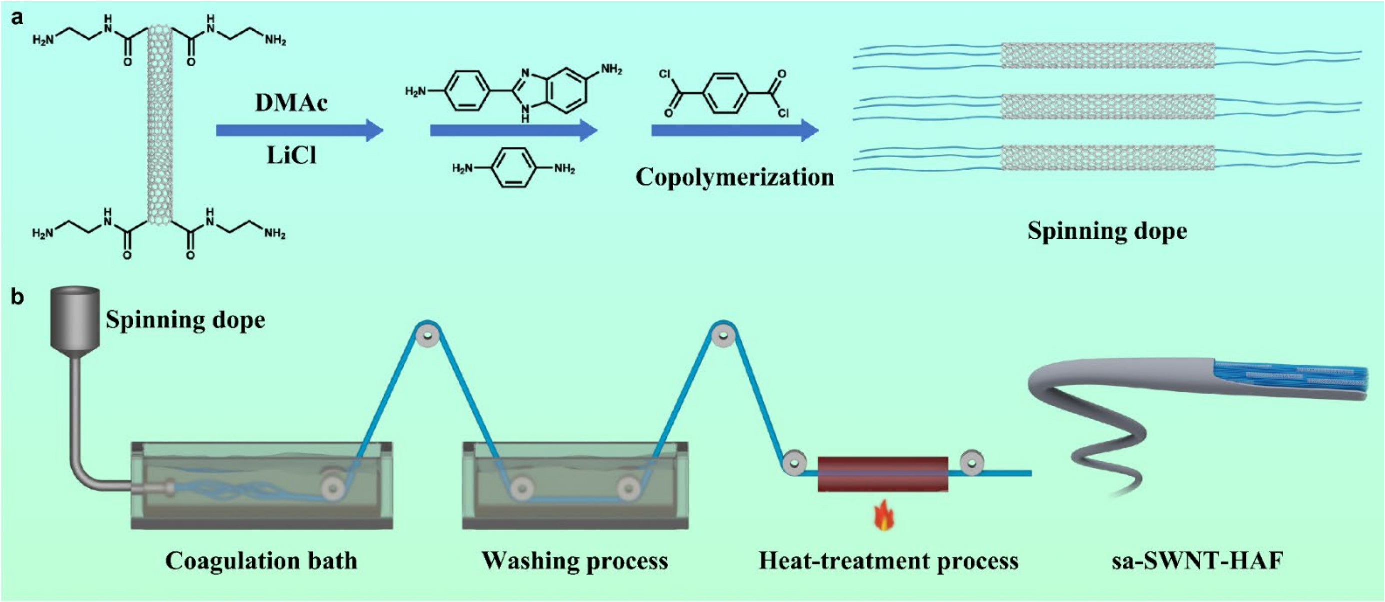

Supplementary Fig. 6 | Schematic diagram of the fabrication steps of fibers. a, Schematic diagram of preparation of spinning dope. **b,** Schematic diagram of wet spinning.

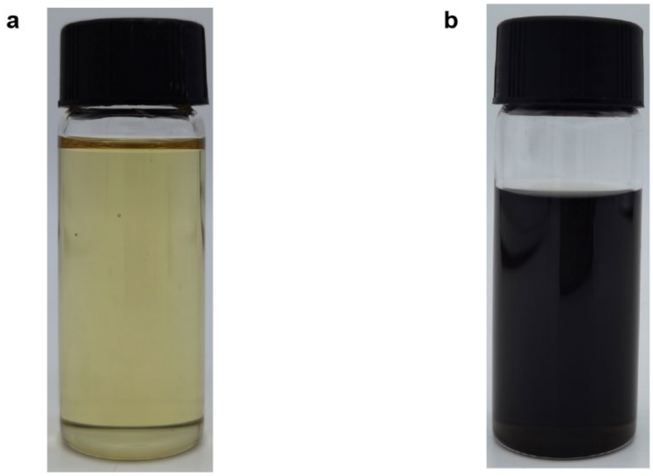

Supplementary Fig. 7 | Spinning dope pictures of (**a**) heterocyclic aramid and (**b**) 0.05 wt% sa-SWNT/heterocyclic aramid.

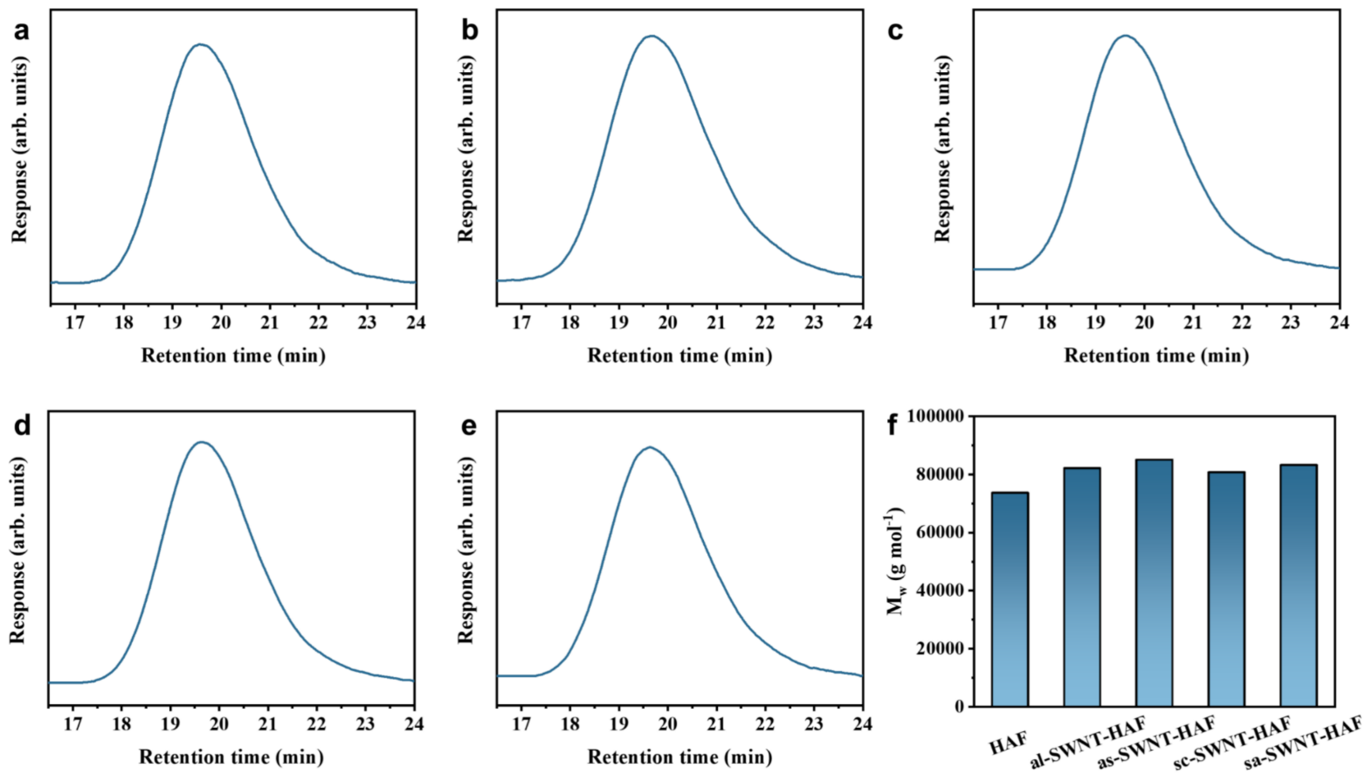

Supplementary Fig. 8 | GPC tests of different polymer solutions. Chromatogram of polymer solutions of (**a**) HAF, (**b**) al-SWNT-HAF, (**c**) as-SWNT-HAF, (**d**) sc-SWNT-HAF, and (**e**) sa-SWNT-HAF. **f**, The weight-average molecular weight comparison of different polymer solutions. In GPC testing, due to the filtration of solutions before testing and the low concentration of SWNTs (0.05 wt%) in polymer solutions, the molecular weight of polymer chains covalently bonding with SWNTs is not available. Thus, all the GPC results reflect the molecular weight of pure heterocyclic aramid polymer chains in different solutions.

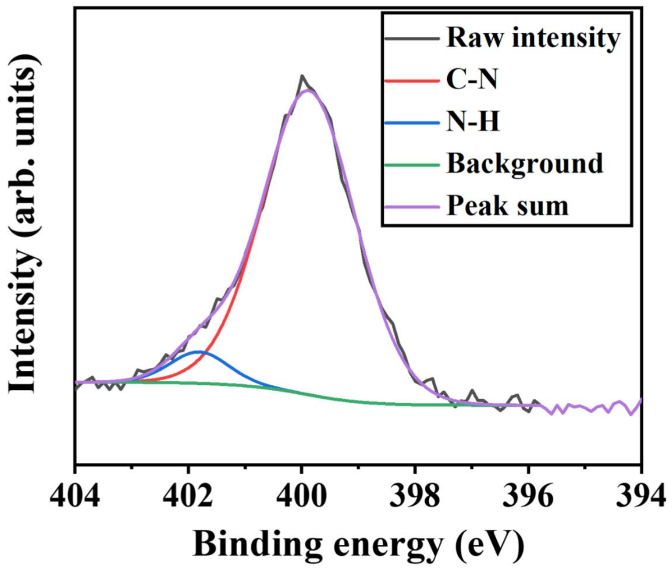

Supplementary Fig. 9 | XPS high-resolution N *1s* spectra of the product from the reactions of sa-SWNTs and TPC.

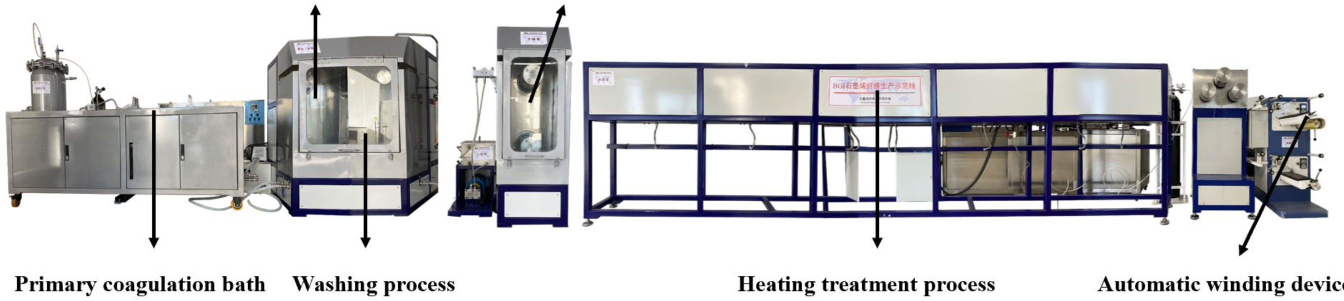

Supplementary Fig. 10 | Picture of spinning equipment.

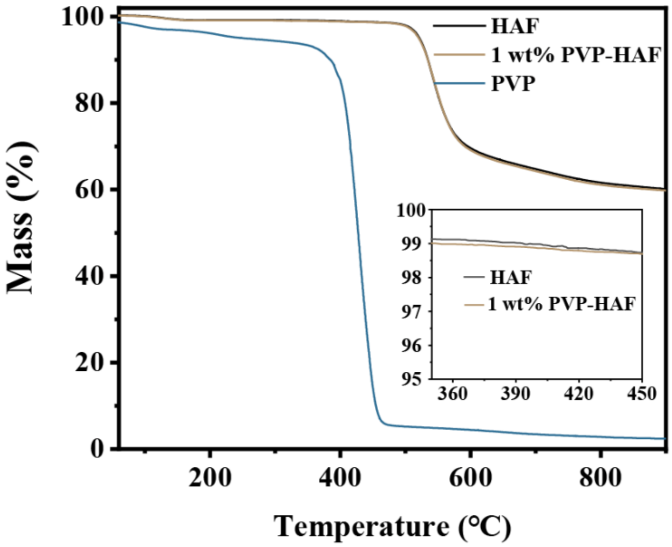

Supplementary Fig. 11 | Thermogravimetric curves of HAFs, 1 wt% PVP-HAFs, and PVP.

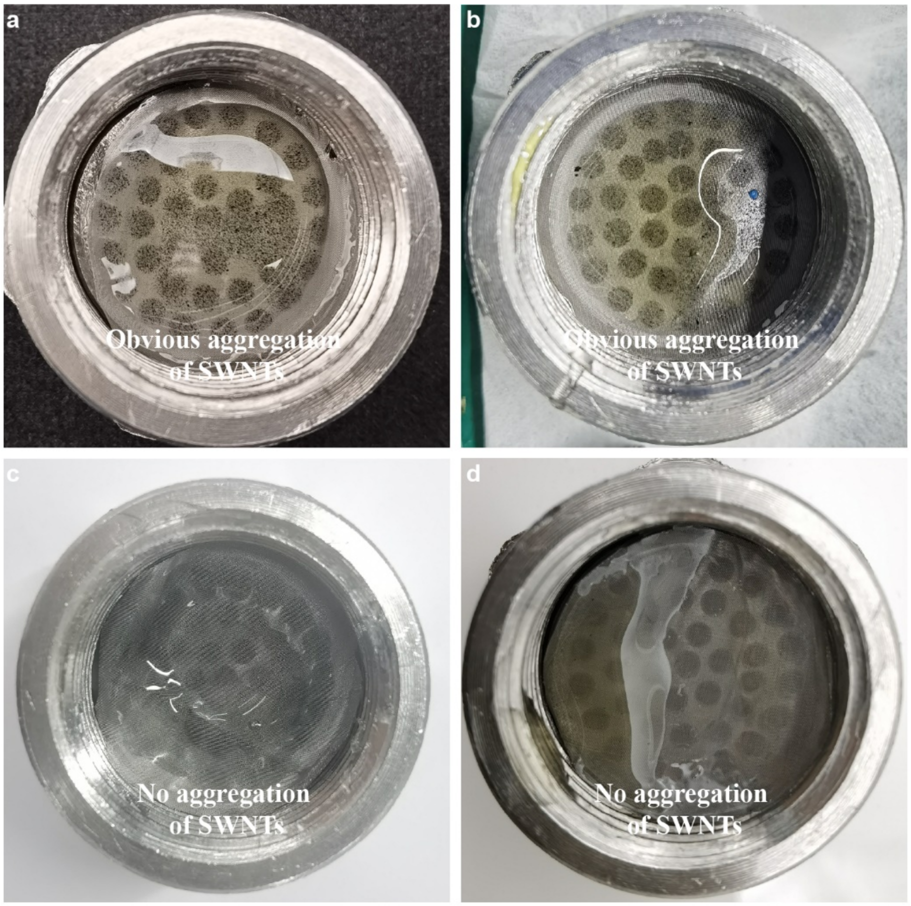

Supplementary Fig. 12 | Spinnability characterization of pinning dopes after six hours of wet spinning process with the addition of (**a**) al-SWNTs, (**b**) as-SWNTs, (**c**) sc-SWNTs, and (**d**) sa-SWNTs.

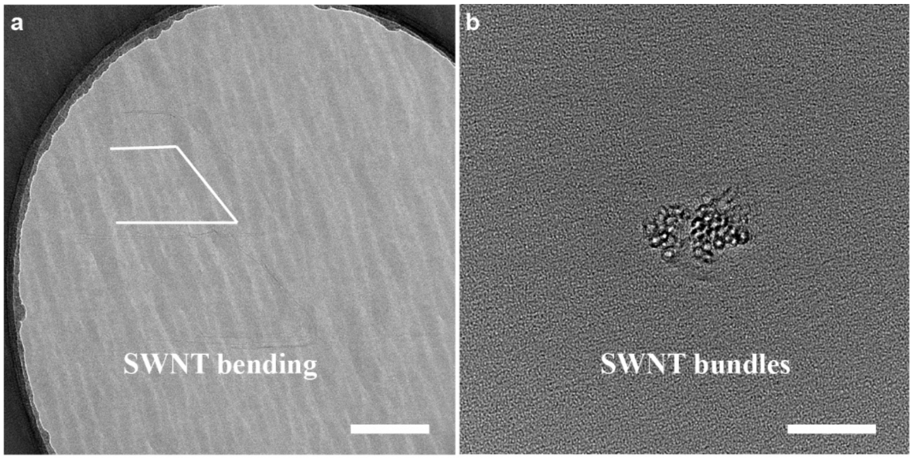

Supplementary Fig. 13 | TEM images of the axial cross-section and radial cross-section of al-SWNT-HAFs. **a,** TEM image of the radial cross-section of al-SWNT-HAFs prepared by embedded ultramicrotome. Scale bar, 100 nm. **b,** TEM image of the axial cross-section of al-SWNT-HAFs prepared by FIB. Scale bar, 20 nm.

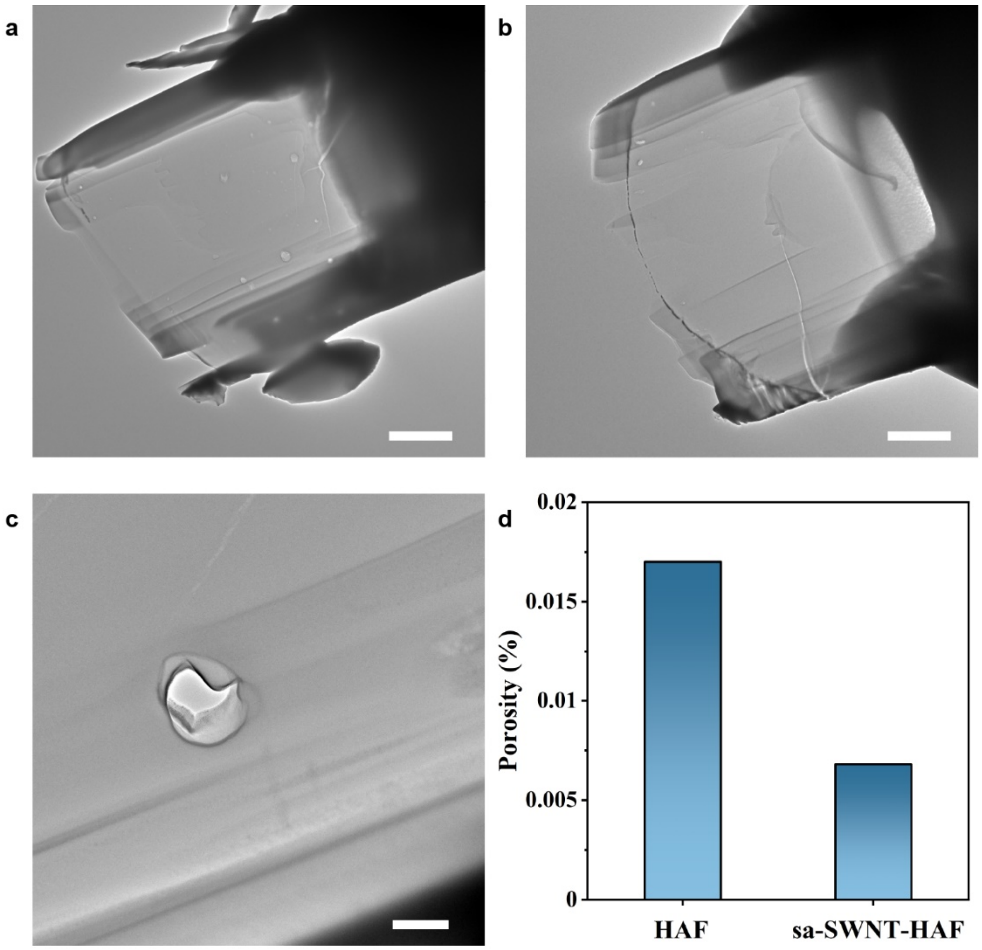

Supplementary Fig. 14 | Calculation of porosities of HAFs and sa-SWNT-HAFs. TEM images of (**a**) HAFs and (**b**) sa-SWNT-HAFs prepared by FIB. **c,** TEM image of pore morphology prepared by FIB for HAFs. Scale bars, 2 μm in **a, b**; 200 nm in **c**. **d,** Porosities of HAFs and sa-SWNT-HAFs derived from FIB-SEMT.

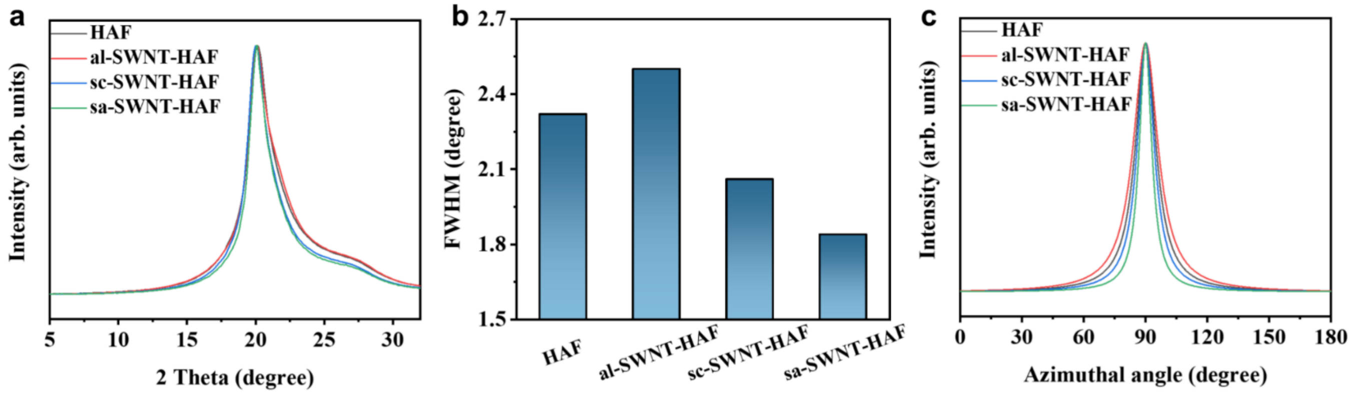

Supplementary Fig. 15 | 2D-WAXS analysis of different fibers. a, One-dimensional WAXS (1D-WAXS) curves of different fibers from 2D-WAXS analysis. **b,** Comparison of the FWHM of different fibers from the 1D-WAXS curves. **c,** The azimuthal intensity profile of different fibers from the 2D-WAXS analysis. The lines were achieved by Lorentz fitting.

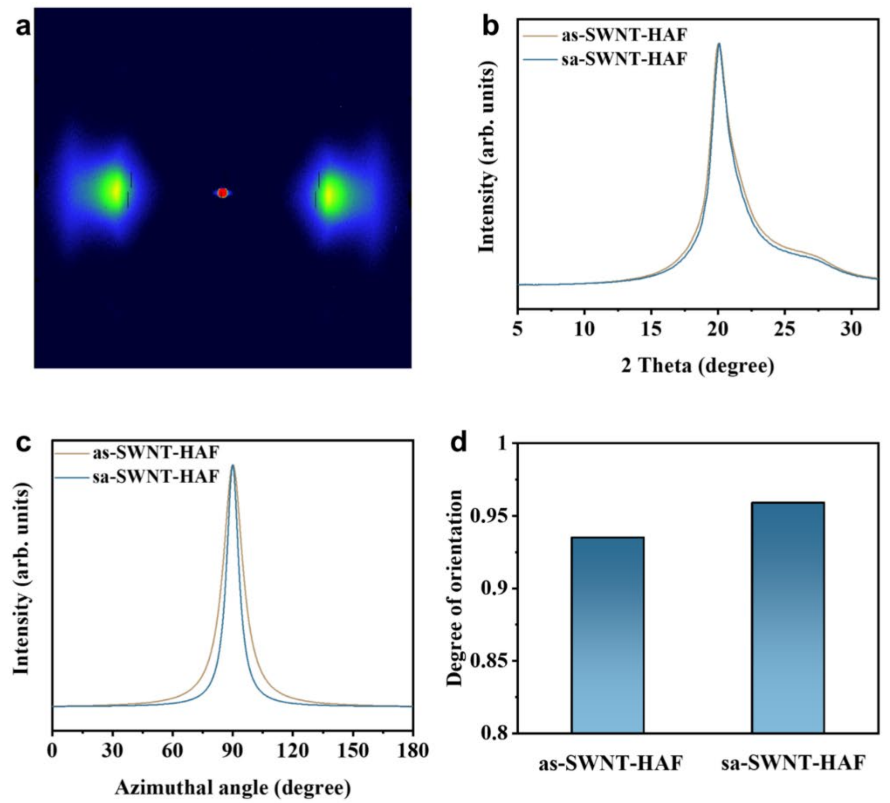

Supplementary Fig. 16 | 2D-WAXS analysis of as-SWNT-HAFs and sa-SWNT-HAFs. a, 2D-WAXS patterns of as-SWNT-HAFs. **b,** Comparison of the 1D-WAXS curves of as-SWNT-HAFs and sa-SWNT-HAFs from 2D-WAXS analysis. **c,** The azimuthal intensity profiles of as-SWNT-HAFs and sa-SWNT-HAFs from the 2D-WAXS analysis. The lines were achieved by Lorentz fitting. **d,** Comparison of the degree of orientation of as-SWNT-HAFs and sa-SWNT-HAFs from 2D-WAXS analysis.

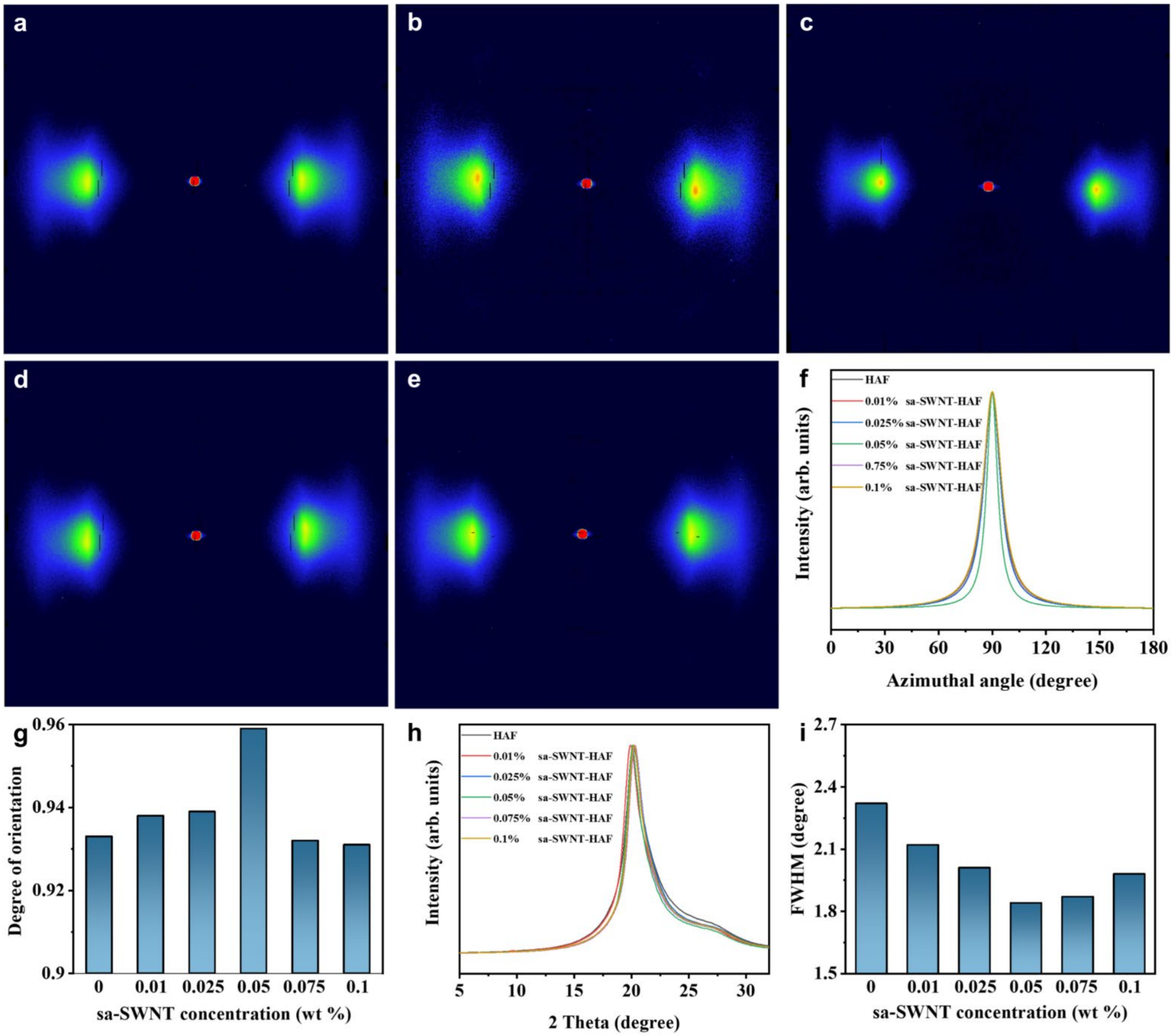

Supplementary Fig. 17 | 2D-WAXS analysis of sa-SWNT-HAFs with different concentrations of sa-SWNTs. 2D-WAXS patterns of sa-SWNT-HAFs with (**a**) 0.01%, (**b**) 0.025%, (**c**) 0.05%, (**d**) 0.075%, (**e**) 0.1% concentration of sa-SWNTs. **f,** The azimuthal intensity profile of sa-SWNT-HAFs with different concentrations of sa-SWNTs from the 2D-WAXS analysis. The lines were achieved by Lorentz fitting. **g,** Comparison of the degree of orientation of sa-SWNT-HAFs with different concentrations from the 2D-WAXS analysis. **h,** Comparison of the 1D-WAXS curves with different concentrations of sa-SWNT-HAFs from the 2D-WAXS analysis. **i,** Comparison of the FWHM with different concentrations of sa-SWNT-HAFs from the 1D-WAXS curves.

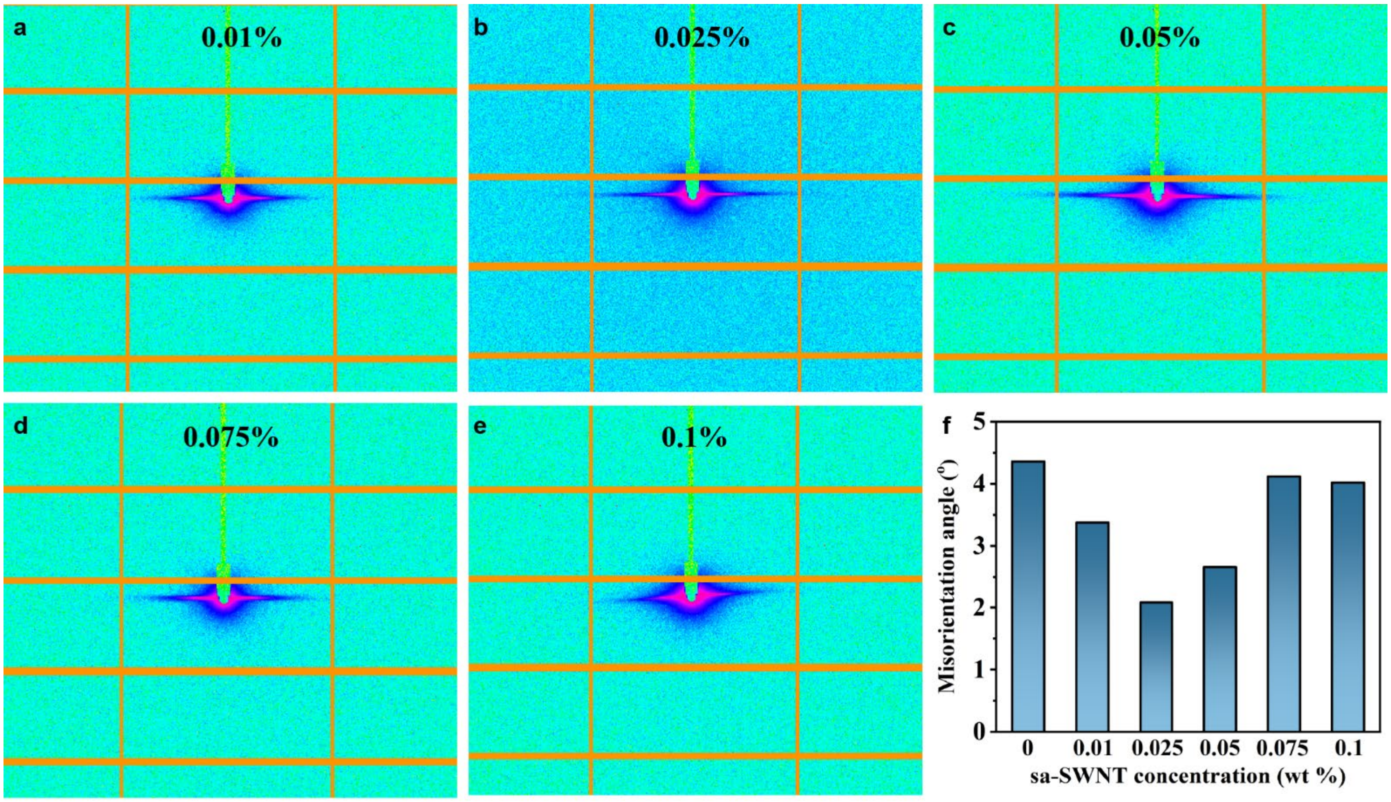

Supplementary Fig. 18 | 2D-SAXS analysis of sa-SWNT-HAFs with different concentrations of sa-SWNTs. 2D-SAXS patterns of sa-SWNT-HAFs with (**a**) 0.01%, (**b**) 0.025%, (**c**) 0.05%, (**d**) 0.075%, (**e**) 0.1% concentration of sa-SWNTs. **f,** Comparison of the microfibril misorientation degree with different concentrations of sa-SWNT-HAFs from 2D-SAXS analysis.

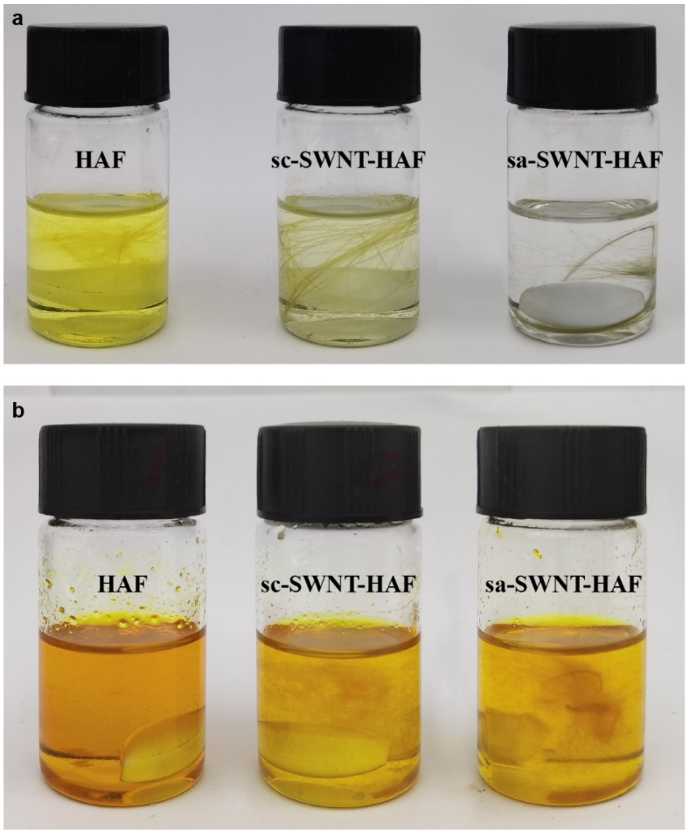

Supplementary Fig. 19 | Digital photographs of different fibers dissolved in KOH/DMSO solution at different times. HAFs, sc-SWNT-HAFs, and sa-SWNT-HAFs dissolved in KOH/DMSO solution for (**a**) 1 hour and (**b**) 12 hours.

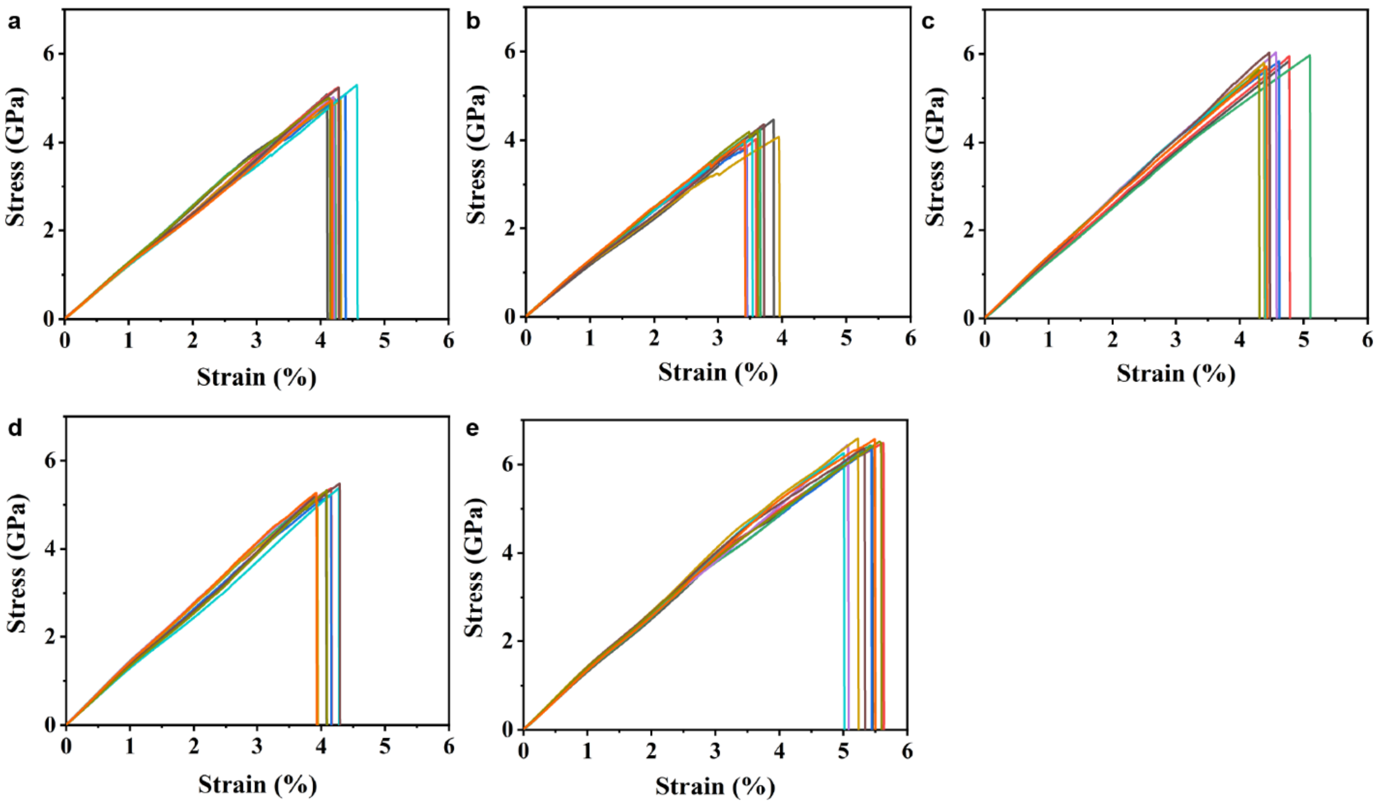

Supplementary Fig. 20 | Stress-strain curves of different fibers. Stress-strain curves of (**a**) HAFs, (**b**) al-SWNT-HAFs, (**c**) as-SWNT-HAFs, (**d**) sc-SWNT-HAFs, and (**e**) sa-SWNT-HAFs. The concentration of SWNTs is 0.05 wt%.

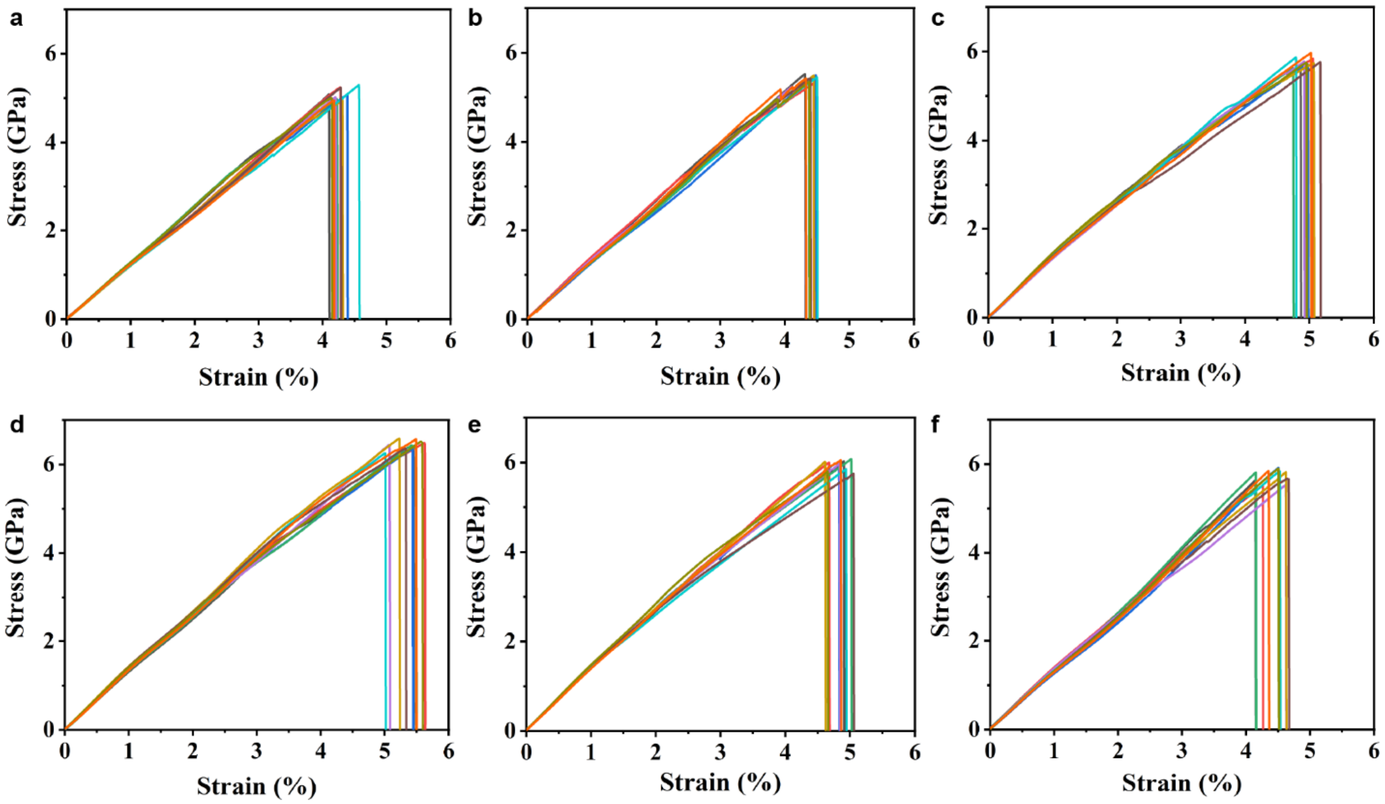

Supplementary Fig. 21 | Stress-strain curves of sa-SWNT-HAFs with different concentrations. Stress-strain curves of sa-SWNT-HAFs with concentrations of (**a**) 0.00 wt%, (**b**) 0.01 wt%, (**c**) 0.025 wt%, (**d**) 0.05 wt%, (**e**) 0.075 wt%, and (**f**) 0.1 wt% sa-SWNTs.

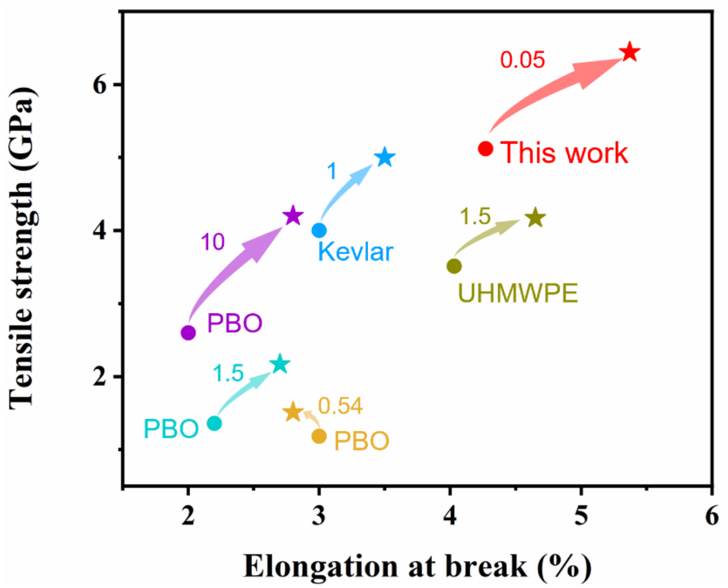

Supplementary Fig. 22 | Strength and elongation at break of typical high-performance fibers reinforced by CNTs. The data, which are shown in **Supplementary Table S10**, are taken from the literature.

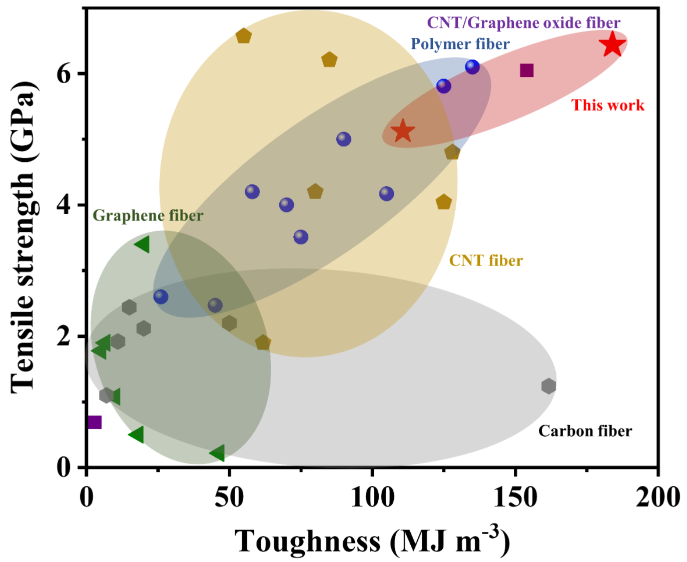

Supplementary Fig. 23 | Tensile strength versus toughness of our fibers, carbon fibers, graphene fibers, CNT fibers, CNT/graphene oxide fibers, and polymer fibers. The triangle, pentagon, hexagon, square, and circle represent the data of graphene fibers, CNT fibers, carbon fibers, CNT/graphene oxide fibers, and polymer fibers, respectively. The raw data, which are shown in **Supplementary Table 12**, are taken from the literature.

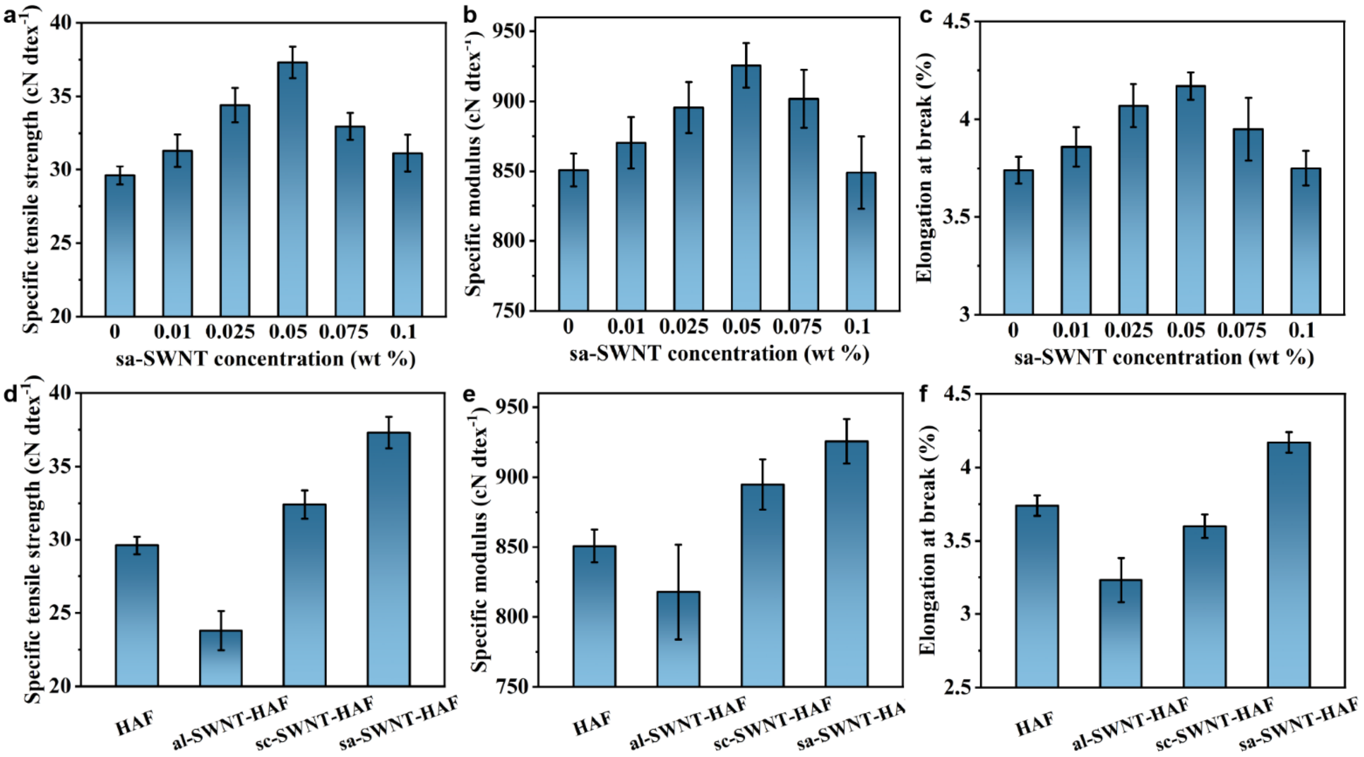

Supplementary Fig. 24 | Mechanical properties of composite fiber yarns. Comparison of (**a**) the specific tensile strength, (**b**) the specific modulus, and (**c**) the elongation at break of sa-SWNT-HAF yarns with different concentrations of sa-SWNTs. Comparison of (**d**) the specific tensile strength, (**e**) the specific modulus, and (**f**) the elongation at break of different fiber yarns. Error bars indicate the standard deviation.

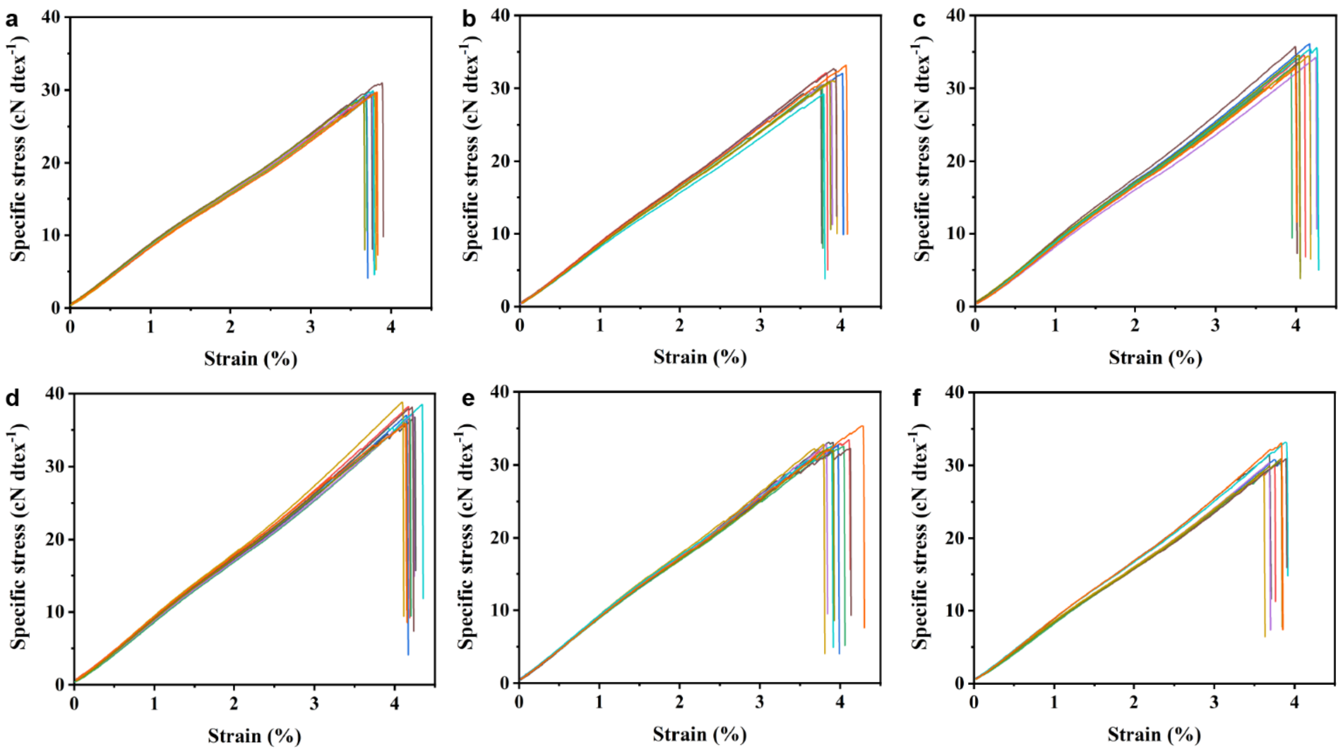

Supplementary Fig. 25 | Specific stress-strain curves of sa-SWNT-HAF yarns with different concentrations. Specific stress-strain curves of sa-SWNT-HAF yarns with (**a**) 0.00 wt%, (**b**) 0.01 wt%, (**c**) 0.025 wt%, (**d**) 0.05 wt%, (**e**) 0.075 wt%, and (**f**) 0.1 wt% concentration of sa-SWNTs.

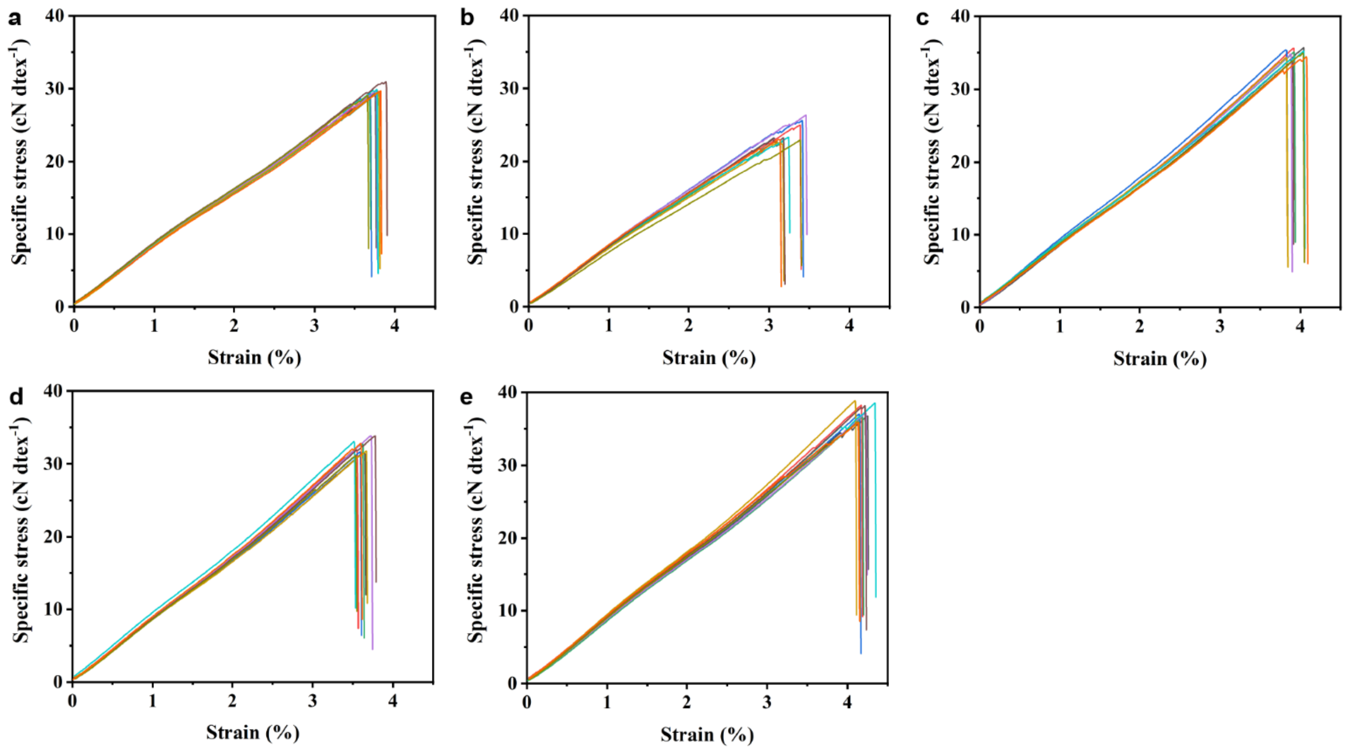

Supplementary Fig. 26 | Specific stress-strain curves of different fiber yarns. Specific stress-strain curves of (**a**) HAF yarns, (**b**) al-SWNT-HAF yarns, (**c**) sc-SWNT-HAF yarns, (**d**) as-SWNT-HAF yarns, and (**e**) sa-SWNT-HAF yarns. The concentration of SWNTs is 0.05 wt%.

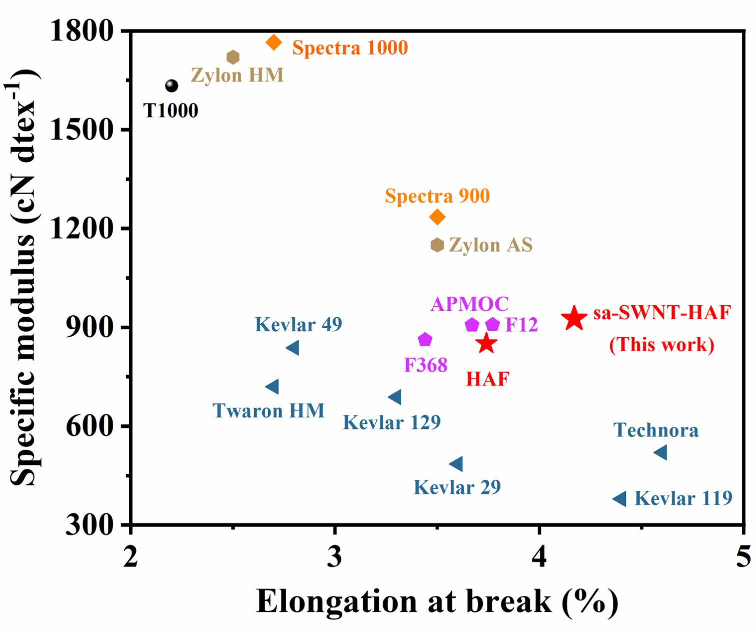

Supplementary Fig. 27 | Specific modulus and elongation at break of various commercial high-performance fibers. The data are shown in **Supplementary Table 15**.

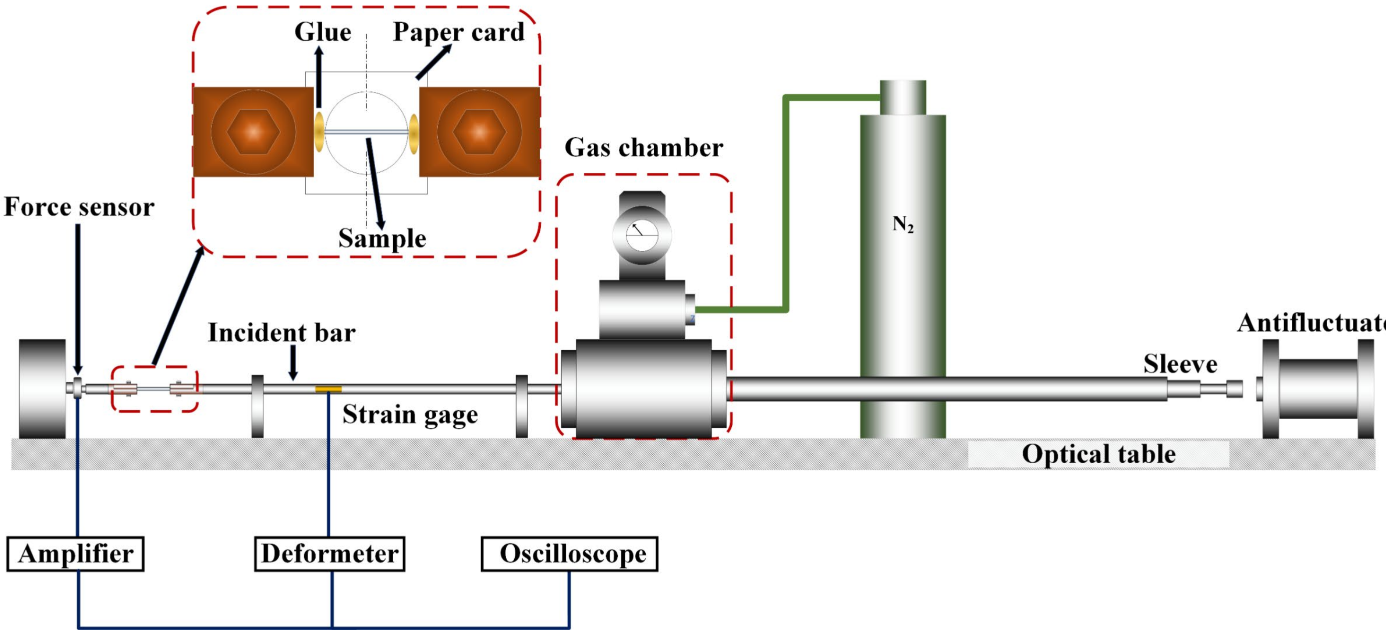

Supplementary Fig. 28 | Schematic diagram of the mini-SHTB experiment. While a sleeve-typed bullet launched by a gas gun strikes the mass block fixed at the end of the incident bar, a tensile wave is generated and propagates along the incident bar. When it reaches the specimen clamped at the end of the incident bar, the dynamic tension is applied on the single fiber specimen, causing its ultimate failure.

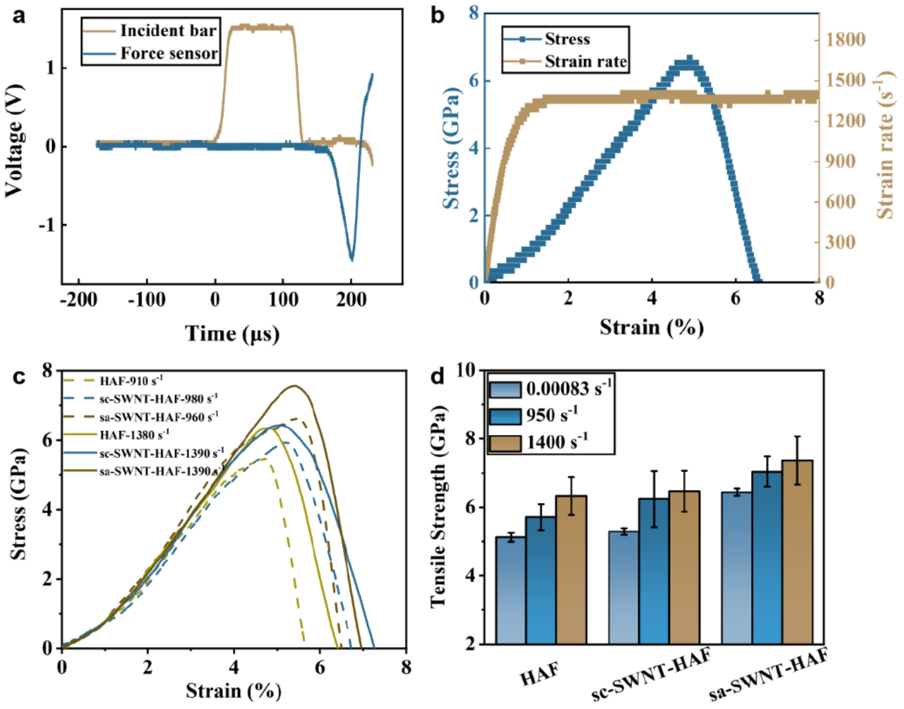

Supplementary Fig. 29 | Analysis of dynamic tensile strength. a, Typical signals of the incident wave and force. **b,** Typical stress-strain and strain rate-strain curve. **c,** Stress-strain curves of HAFs, sc-SWNT-HAFs, and sa-SWNT-HAFs at different strain rates. **d,** Comparison of the tensile strength with different strain rates of HAFs, sc-SWNT-HAFs, and sa-SWNT-HAFs. Error bars indicate the standard deviation of tensile strength.

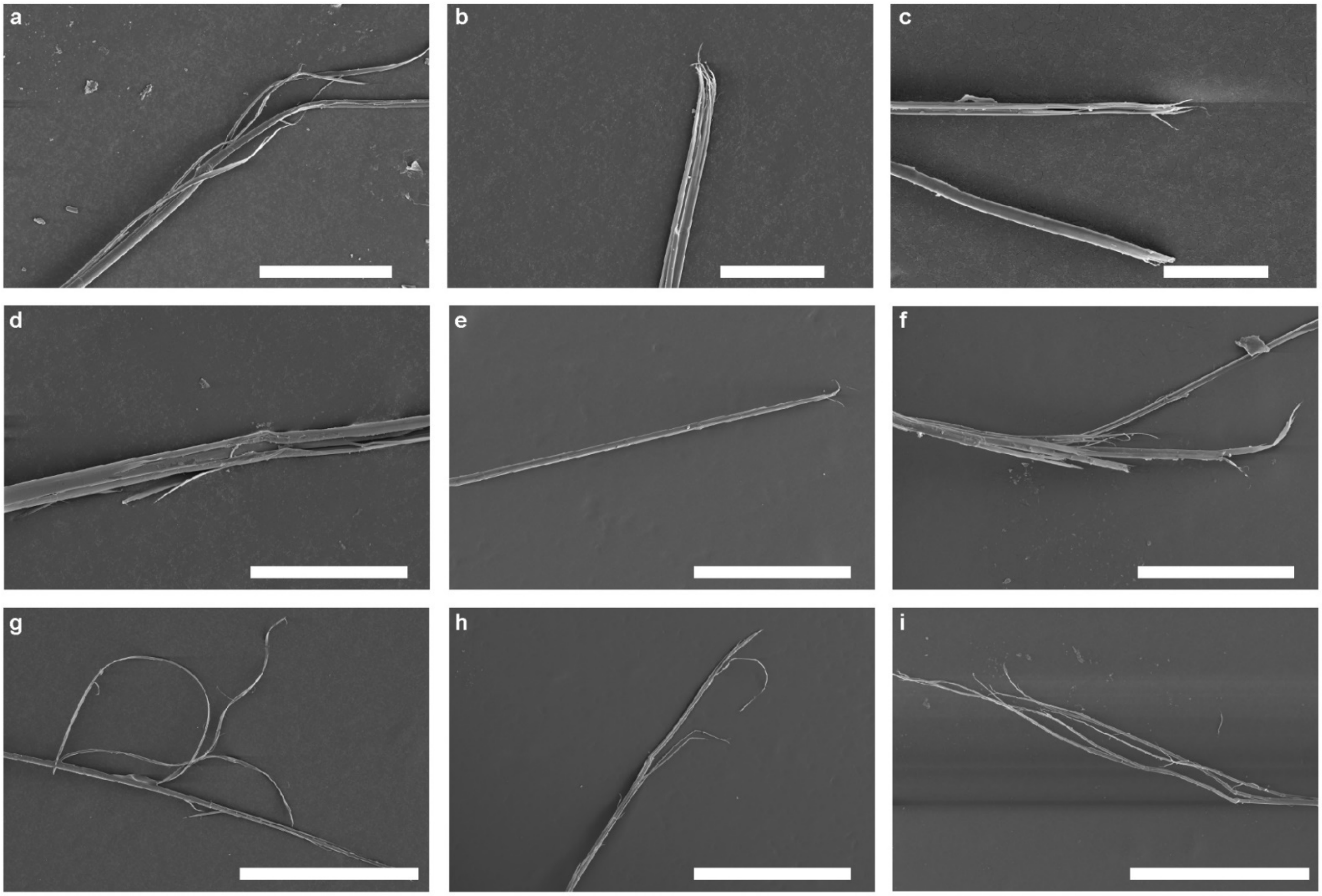

Supplementary Fig. 30 | SEM images of the fracture point of different fibers at different strain rates. SEM images of the fracture point of (**a**) HAFs, (**b**) sc-SWNT-HAFs, and (**c**) sa-SWNT-HAFs at the strain rate of 0.00083 s^{-1}. SEM images of the fracture point of (**d**) HAFs, (**e**) sc-SWNT-HAFs, and (**f**) sa-SWNT-HAFs at the strain rate of 950 s^{-1}. SEM images of the fracture point of (**g**) HAFs, (**h**) sc-SWNT-HAFs, and (**i**) sa-SWNT-HAFs at the strain rate of 1400 s^{-1}. Scale bars, 200 μm in **a, f**; 50 μm in **b, c**; 100 μm in **d**; 300 μm in **e**; 500 μm in **g, h, i**.

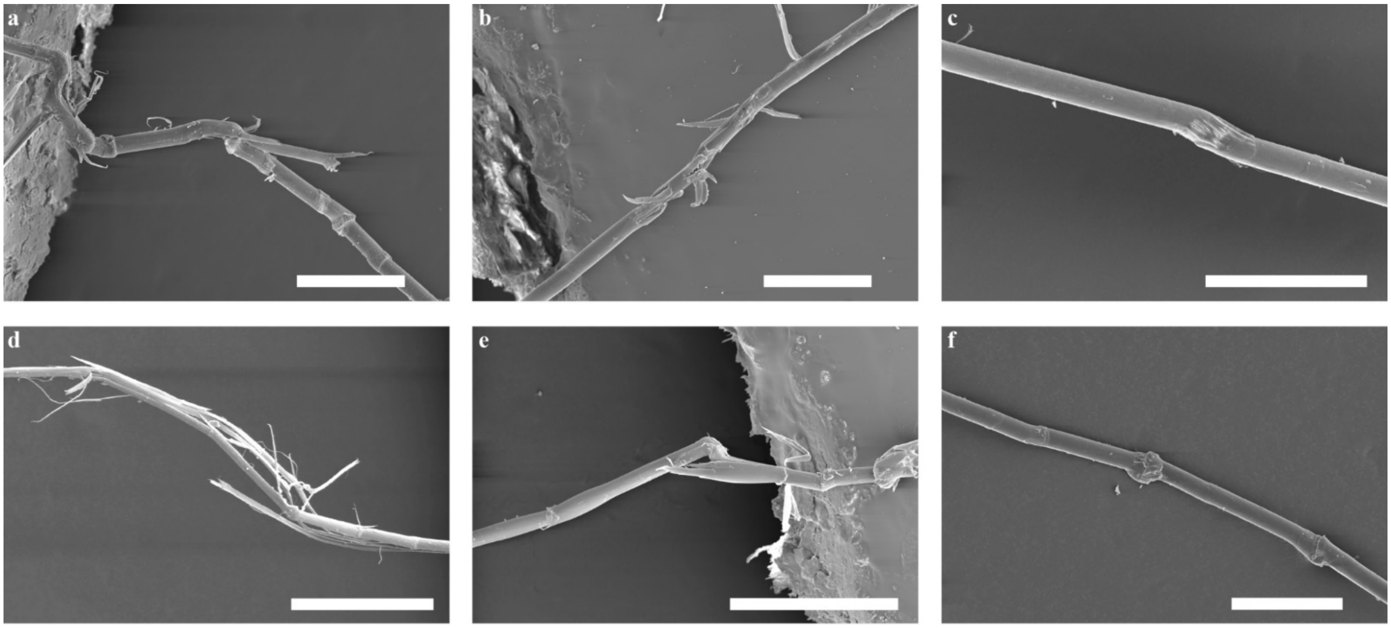

Supplementary Fig. 31 | SEM images of the kink bond of different fibers at different strain rates. SEM images of the kink bond of (**a**) HAFs, (**b**) sc-SWNT-HAFs, and (**c**) sa-SWNT-HAFs at the strain rate of 950 s^{-1}. SEM images of the kink bond of (**d**) HAFs, (**e**) sc-SWNT-HAFs, and (**f**) sa-SWNT-HAFs at the strain rate of 1400 s^{-1}. The kink bond was formed away from the fracture point of fiber, due to the sudden release of stress after fiber tensile fracture in the tensile tests. Scale bars, 200 μm in **a, b, c, f**; 50 μm in **d, e**.

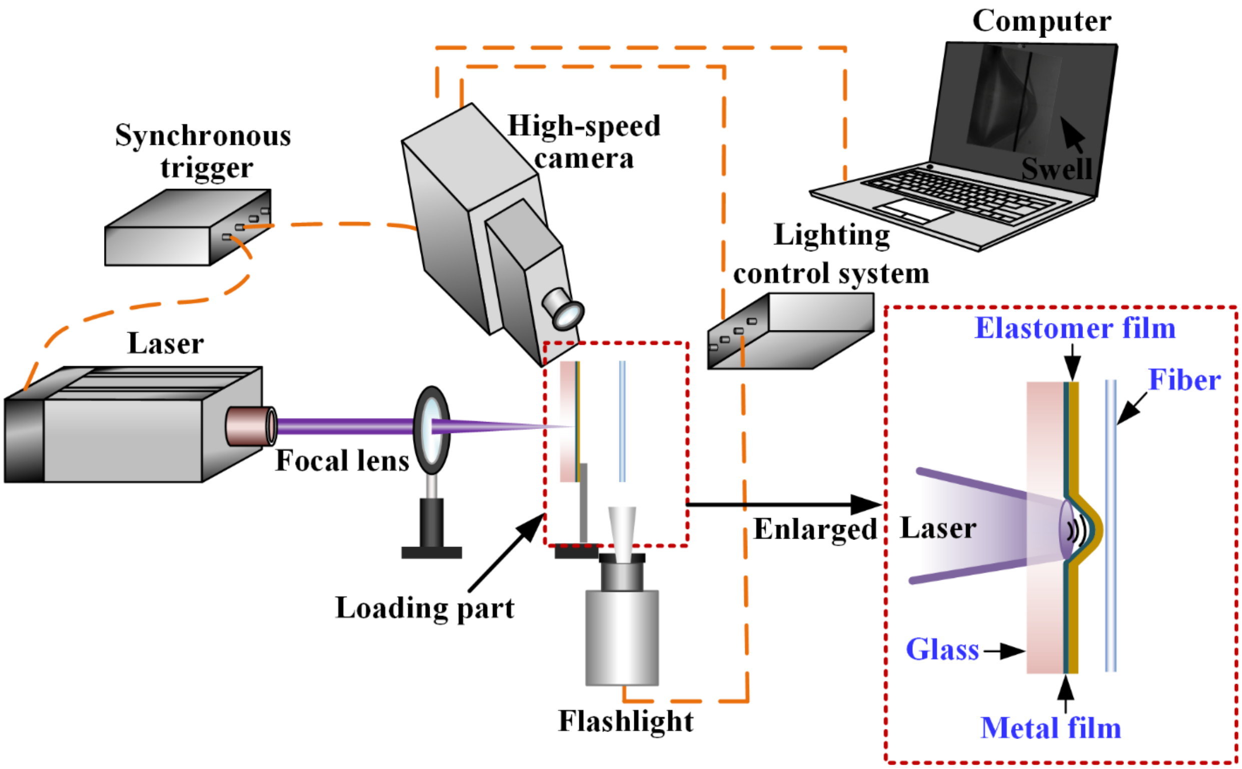

Supplementary Fig. 32 | Schematic of the laser-shock induced polydimethylsiloxane film swelling impact on a single fiber. The laser ablates the 100 nm thick gold film to create a fast expanding plasma on the surface, causing the fast expansion of the 76 μm thick polydimethylsiloxane (PDMS) layer, which impacts the single fiber specimen with high velocity.

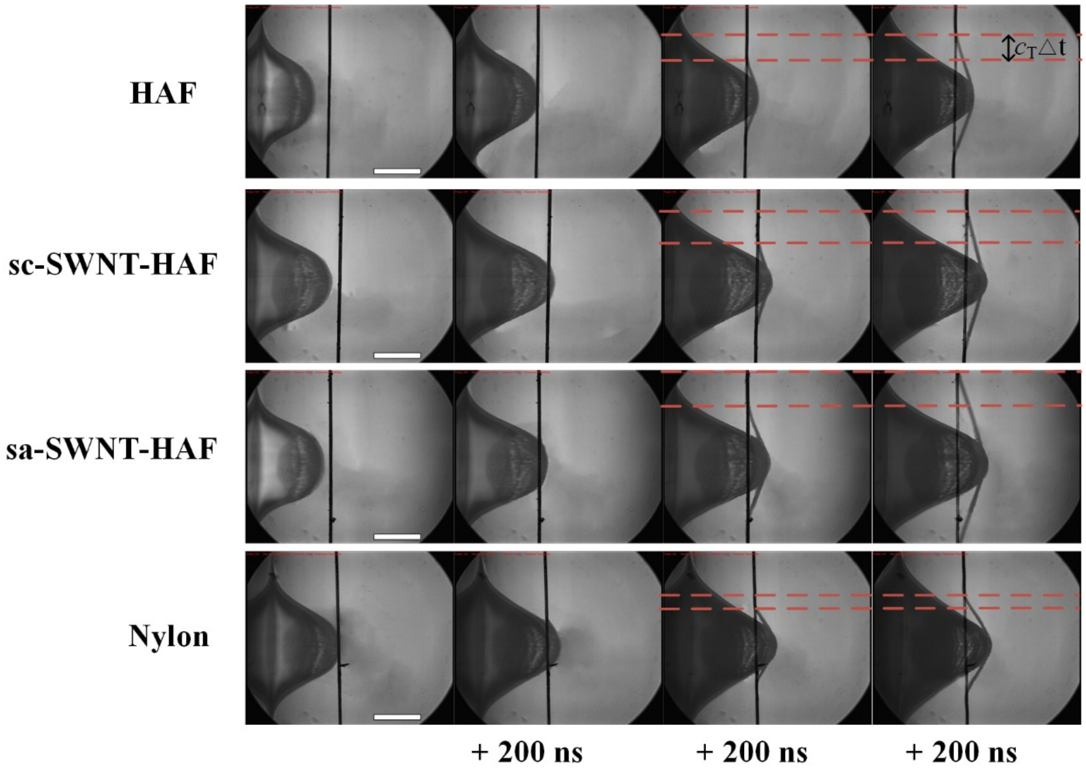

Supplementary Fig. 33 | Deformation process under transverse impact for different fibers. Scale bar, 300 μm.

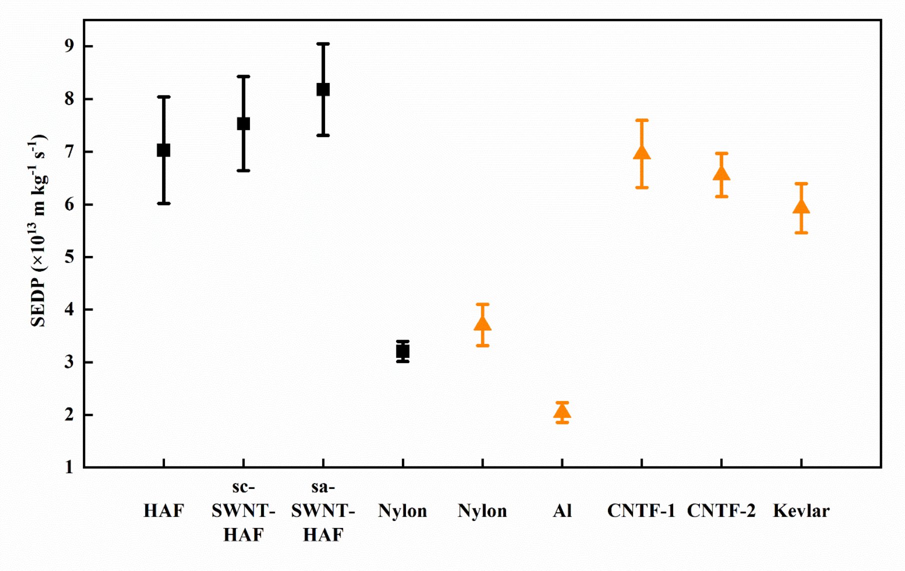

Supplementary Fig. 34 | SEDP of different fibers. The orange dots in the figure are collected from reference[7]. Error bars indicate the standard deviation of SEDP.

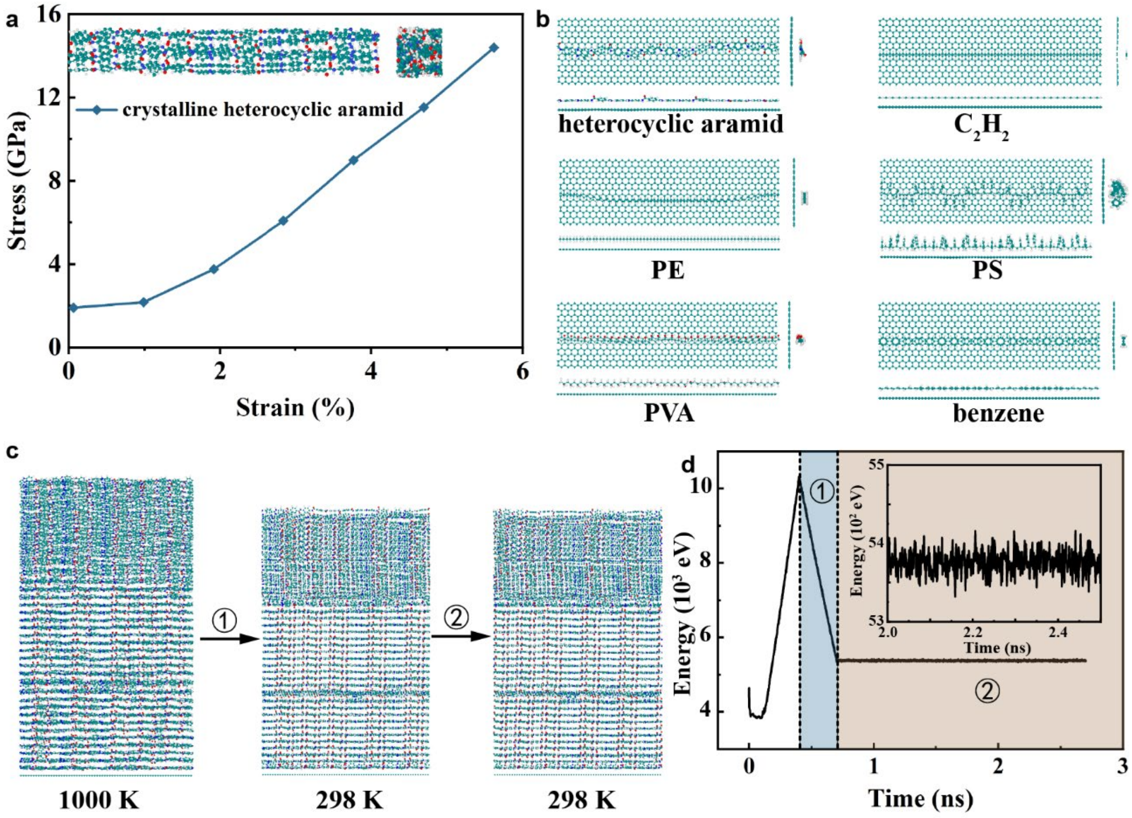

Supplementary Fig. 35 | Theoretical calculations of composite fibers. a, Stress-strain curve of crystalline heterocyclic aramid. **b,** Molecular structure of typical polymer chains onto an *sp*^{2} carbon sheet. **c,** Simulation snapshots of heterocyclic aramid chains onto an *sp*^{2} carbon sheet. **d**, Energy evolution of heterocyclic aramid chains onto an *sp*^{2} carbon sheet. The temperature increases from nearly 0 K to 1000 K and then decreases to 298 K. The temperature is controlled as 298 K for 2 ns.

Supplementary Table 1 | Molecular weight of different polymer solutions determined by GPC method.

Polymer solutions	M_p (g mol^{-1})	M_n (g mol^{-1})	M_w (g mol^{-1})	M_z (g mol^{-1})	Polydispersity
**HAF**	6.83×10^4	2.67×10^4	7.36×10^4	1.39×10^5	2.76
**al-SWNT-HAF**	7.41×10^4	2.75×10^4	8.21×10^4	1.70×10^5	2.98
**as-SWNT-HAF**	7.96×10^4	3.32×10^4	8.50×10^4	1.50×10^5	2.56
**sc-SWNT-HAF**	7.64×10^4	3.23×10^4	8.07×10^4	1.42×10^5	2.50
**sa-SWNT-HAF**	7.68×10^4	3.41×10^4	8.32×10^4	1.46×10^5	2.44

Supplementary Table 2 | Mechanical properties of HAFs.

Number	Tensile strength (GPa)	Modulus (GPa)	Elongation at break (%)	Toughness (MJ m^{-3})
**1**	5.20	128.3	4.10	105.8
**2**	5.24	121.6	4.28	116.9
**3**	5.08	125.7	4.39	114.4
**4**	5.21	130.3	4.23	111.2
**5**	5.02	120.9	4.20	106.9
**6**	4.95	121.1	4.31	111.0
**7**	5.29	122.4	4.57	108.0
**8**	5.24	127.6	4.29	121.8
**9**	5.00	128.6	4.14	107.7
**10**	4.95	124.4	4.18	103.5
**Average**	5.12	125.1	4.27	110.7

Supplementary Table 3 | Mechanical properties of sa-SWNT-HAFs with 0.05 wt% concentration of sa-SWNTs.

Number	Tensile strength (GPa)	Modulus (GPa)	Elongation at break (%)	Toughness (MJ m^{-3})
**1**	6.4	133.6	5.45	183.9
**2**	6.49	144.4	5.63	199.3
**3**	6.35	143.3	5.44	184.2
**4**	6.42	138.1	5.49	186.4
**5**	6.43	143.9	5.08	166.1
**6**	6.59	146.3	5.22	181.5
**7**	6.25	140.4	5.01	165.1
**8**	6.36	145.4	5.34	183.4
**9**	6.51	142.9	5.59	197.3
**10**	6.57	138.2	5.49	192.7
**Average**	6.44	141.7	5.37	184.0

Supplementary Table 4 | Mechanical properties of sa-SWNT-HAFs with 0.01 wt% concentration of sa-SWNTs.

Number	Tensile strength (GPa)	Modulus (GPa)	Elongation at break (%)	Toughness (MJ m^{-3})
**1**	5.52	134.7	4.31	122.7
**2**	5.31	140.5	4.4	129.7
**3**	5.49	129.3	4.48	123
**4**	5.42	131.2	4.38	121.2
**5**	5.33	137.4	4.34	119.9
**6**	5.49	130.2	4.45	126.5
**7**	5.45	126.7	4.5	126
**8**	5.43	132.5	4.4	123.7
**9**	5.34	130.3	4.38	121.5
**10**	5.43	134.1	4.33	122.3
**Average**	5.42	132.7	4.40	123.7

Supplementary Table 5 | Mechanical properties of sa-SWNT-HAFs with 0.025 wt% concentration of sa-SWNTs.

Number	Tensile strength (GPa)	Modulus (GPa)	Elongation at break (%)	Toughness (MJ m^{-3})
**1**	5.64	141.7	4.86	149
**2**	5.84	141.4	5.06	160.6
**3**	5.71	139	4.99	152.8
**4**	5.63	143.3	4.75	143.5
**5**	5.77	133.7	4.92	151.5
**6**	5.74	139.4	5.07	160.3
**7**	5.87	136.7	4.79	146.3
**8**	5.76	135.5	5.17	158.5
**9**	5.75	143.6	4.95	154.7
**10**	5.97	133.5	5.02	156.3
**Average**	5.77	138.8	4.96	153.4

Supplementary Table 6 | Mechanical properties of sa-SWNT-HAFs with 0.075 wt% concentration of sa-SWNTs.

Number	Tensile strength (GPa)	Modulus (GPa)	Elongation at break (%)	Toughness (MJ m^{-3})
**1**	6.02	140.2	4.9	155.5
**2**	6	139	4.68	146.9
**3**	5.99	145.4	4.83	152.9
**4**	6.07	140.1	5.02	162.7
**5**	5.97	144.2	4.84	153
**6**	6.01	142.7	4.61	142.7
**7**	5.85	141	4.94	152.5
**8**	5.75	142.8	5.06	159.2
**9**	5.8	146.2	4.66	146.4
**10**	6.05	141.7	4.85	154.1
**Average**	5.95	142.3	4.84	152.6

Supplementary Table 7 | Mechanical properties of sa-SWNT-HAFs with 0.1 wt% concentration of sa-SWNTs.

Number	Tensile strength (GPa)	Modulus (GPa)	Elongation at break (%)	Toughness (MJ m^{-3})
**1**	5.63	137.4	4.14	116.6
**2**	5.52	140.9	4.26	120.9
**3**	5.91	129	4.51	129.4
**4**	5.81	135.3	4.15	117.9
**5**	5.53	141.4	4.62	132.7
**6**	5.82	133.4	4.63	136.9
**7**	5.83	132.4	4.53	134.6
**8**	5.67	134.3	4.66	137.9
**9**	5.89	132.8	4.51	133.3
**10**	5.85	130	4.35	123.3
**Average**	5.75	134.7	4.43	128.3

Supplementary Table 8 | Mechanical properties of al-SWNT-HAFs with 0.05 wt% concentration of al-SWNTs.

Number	Tensile strength (GPa)	Modulus (GPa)	Elongation at break (%)	Toughness (MJ m^{-3})
**1**	4.46	117.3	3.87	85.2
**2**	4.02	127.2	3.59	76.7
**3**	3.81	127.3	3.45	70.6
**4**	4.28	126.7	3.66	81.0
**5**	3.97	125.7	3.44	71.6
**6**	4.07	120.8	3.95	86.4
**7**	4.04	124.6	3.54	75.3
**8**	4.36	122.8	3.71	80.8
**9**	4.22	125.3	3.62	80.6
**10**	4.00	126.6	3.42	72.0
**Average**	4.12	124.4	3.63	78.0

Supplementary Table 9 | Mechanical properties of sc-SWNT-HAFs with 0.05 wt% concentration of sc-SWNTs.

Number	Tensile strength (GPa)	Modulus (GPa)	Elongation at break (%)	Toughness (MJ m^{-3})
**1**	5.24	146.9	4.07	112.8
**2**	5.37	143.7	4.16	117.0
**3**	5.23	138.3	4.15	113.2
**4**	5.18	143.8	3.94	105.8
**5**	5.25	145.3	3.93	106.8
**6**	5.14	143.8	3.94	105.2
**7**	5.38	129.2	4.28	114.5
**8**	5.48	139.1	4.28	121.0
**9**	5.33	134.9	4.09	108.6
**10**	5.27	143.7	3.92	106.6
**Average**	5.29	140.9	4.08	111.2

Supplementary Table 10 | Mechanical properties of as-SWNT-HAFs with 0.05 wt% concentration of as-SWNTs.

Number	Tensile strength (GPa)	Modulus (GPa)	Elongation at break (%)	Toughness (MJ m^{-3})
**1**	5.85	133.9	4.78	143.8
**2**	5.95	132.5	4.77	145.7
**3**	5.84	137.5	4.61	142
**4**	5.98	128.5	5.1	159
**5**	6.04	140.6	4.56	142.1
**6**	5.8	140	4.38	130.4
**7**	5.7	140.8	4.39	130.8
**8**	6.03	132.8	4.46	135.7
**9**	5.63	142.7	4.3	123.8
**10**	5.72	141	4.43	129.9
**Average**	5.85	137.0	4.58	138.3

Supplementary Table 11 | Strength and elongation at break of typical high-performance fibers reinforced by CNTs.

Materials	CNT wt %	Strength (GPa)	Elongation at break (%)	Ref.
Kevlar	--	4	3	8
Kevlar-CNT	1	5	3.5	8
PBO-1	--	2.6	2	9
PBO-1-CNT	10	4.2	2.8	9
PBO-2	--	1.18	3	10
PBO-2-CNT	0.54	1.51	2.8	10
PBO-3	--	1.36	2.2	11
PBO-3-CNT	1.5	2.17	2.7	11
UHMWPE	--	3.51	4.03	12
UHMWPE-CNT	5	4.17	4.65	12
**HAF (This work)**	--	**5.12**	**4.27**	--
**sa-SWNT-HAF (This work)**	**0.05**	**6.44**	**5.37**	--

Supplementary Table 12 | Comparisons of mechanical properties of our fibers, carbon fibers, graphene fibers, CNT fibers, and polymer fibers (mechanical properties were collected for monofilaments).

Materials	Strength (GPa)	Modulus (GPa)	Elongation at break (%)	Toughness (MJ m^{-3})	Density (g cm^{-3})	Gauge length (mm)	Loading rate (mm min^{-1})	Ref.
Kevlar	4	120	3	~70	1.50	--	15	8
Kevlar-CNT	5	130	3.5	~90	1.50	--	15	8
PBO	2.6	138	2	~26	--	25.4	5	9
PBO-CNT	4.2	167	2.8	~58	--	25.4	5	9
UHMWPE	3.51	122.6	4.03	~75	0.97	--	2.54	12
UHMWPE-CNT	4.17	136.8	4.65	~105	0.995	--	2.54	12
Kevlar 29	2.47	84.5	3.2	~ 45	1.45	30	4.07	13
Heterocyclic aramid fiber[1]	6.1	150.1	4.35	~135	--	20	10	3
Heterocyclic aramid fiber[2]	5.81	143.2	4.15	~125	--	20	1	14
Graphene fiber[1]	0.22	--	39	~46.3	0.8	7	0.06	15
Graphene fiber[2]	0.501	11.2	6.7	~18	--	--	10% min^{-1}	16
Graphene fiber[3]	1.78	385	0.5	~5	--	5	10% min^{-1}	17
Graphene fiber[4]	1.9	309	0.67	~6.5	--	20	0.5	18
Graphene fiber[5]	3.4	341.7	1	~20	1.9	5	--	19

Graphene fiber[6]	1.08	77.6	1.45	~10	1.74	--	0.5	20
CNT fiber[1]	4.2	260	3.5	~80	--	--	--	21
CNT fiber[2]	1.9	195	5.28	~61.8	0.2	10	3	22
CNT fiber[3]	4.04	83.3	6.01	~125	--	10	0.6	23
CNT fiber[4]	6.57	629	1.53	~55	1.71	25	--	24
CNT-PI fiber	4.8	390	4.1	~128	1.78	25	2	25
	6.21	528	1.69	~85	1.74	25	2	25
Carbon fiber	1.24	13.5	28	~161.7	1.18	10	5	26
Graphene/Carbon fiber[1]	2.44	358.3	0.7	~15	1.9	5	--	27
Graphene/Carbon fiber[2]	1.92	233	1.1	~11	~1.6	20	10^{-4}	28
Graphene oxide/Carbon fiber[1]	1.1	100	1.27	6.96	1.43-1.69	25	2.5	29
Graphene oxide/Carbon fiber[2]	2.12	138	1.53	~20	1.73	25	2	30
CNT/ Carbon fiber[2]	2.2	60	4	~50	1.48	10	1	31
CNT/Graphene oxide fiber	6.05	422	3.6	154	2.01	25	2	32
CNT/Graphene oxide fiber	0.69	--	0.6	~3	1.6	25	2.5	33

HAF (This work)	5.12	125.1	4.27	110.7	1.44	20	1	--
sa-SWNT-HAF (This work)	6.44	141.7	5.37	184.0	1.45	20	1	--

Supplementary Table 13 | Comparison of the mechanical properties of sa-SWNT-HAF yarns with different concentrations of sa-SWNTs.

The sa-SWNT concentration (wt%)	Linear density (tex)	Tensile strength (cN dtex^{-1})	Elongation at break (%)	Modulus (cN dtex^{-1})
0	5.45	29.60 ± 0.62	3.74 ± 0.07	850.79 ± 11.93
0.01	5.64	31.30 ± 1.12	3.86 ± 0.10	870.44 ± 18.33
0.025	5.48	34.40 ± 1.17	4.07 ± 0.11	895.62 ± 18.19
**0.05**	**5.51**	**37.31 ± 1.07**	**4.17 ± 0.07**	**925.64 ± 15.97**
0.075	5.52	32.96 ± 0.92	3.95 ± 0.16	901.77 ± 20.71
0.1	5.15	31.13 ± 1.28	3.75 ± 0.09	848.92 ± 26.02

Supplementary Table 14 | Comparison of the mechanical properties of different fiber yarns. The addition of corresponding SWNT is 0.05 wt%.

Materials	Linear density (tex)	Tensile strength (cN dtex^{-1})	Elongation at break (%)	Modulus (cN dtex^{-1})
HAF	5.46	29.60 ± 0.62	3.74 ± 0.07	850.79 ± 11.93
al-SWNT-HAF	5.22	23.79 ± 1.34	3.23 ± 0.15	817.87 ± 33.99
as-SWNT-HAF	5.31	34.95 ± 0.58	3.92 ± 0.10	872.61 ± 20.06
sc-SWNT-HAF	5.81	32.41 ± 0.96	3.60 ± 0.08	894.84 ± 17.93
**sa-SWNT-HAF**	**5.51**	**37.31 ± 1.07**	**4.17 ± 0.07**	**925.64 ± 15.97**

Supplementary Table 15 | Mechanical properties of various commercial high-performance fibers[34-37] (Mechanical properties derived from multifilament testing).

Type	Materials	Tensile strength (cN dtex^{-1})	Elongation at break (%)	Modulus (cN dtex^{-1})
p-Aramid	Kevlar 29	20	3.6	485
	Kevlar 49	20	2.8	838
	Kevlar 119	21	4.4	379
	Kevlar 129	23	3.3	688
	Twaron HM	21	2.7	720
	Technora	25	4.6	520
PBO	Zylon AS	37	3.5	1150
	Zylon HM	37	2.5	1720
UHMWPE	Spectra 900	26	3.5	1235
	Spectra 1000	30	2.7	1765
Carbon fiber	T1000G	35.39	2.2	1633
Heterocyclic aramid	F368	29.47	3.44	862.22
	F12	30.55	3.77	907.87
	APMOC	26.57	3.67	906.58
	**HAF**	**29.60**	**3.74**	**850.79**
	**sa-SWNT-HAF**	**37.31**	**4.17**	**925.64**

Supplementary References

1. Wan, S. *et al.*, High-strength scalable MXene films through bridging-induced densification. *Science* **374**, 96-99 (2021).
2. Li, K. *et al.*, Enhancing mechanical properties of aromatic polyamide fibers containing benzimidazole units via temporarily suppressing hydrogen bonding and crystallization. *J. Appl. Polym. Sci.* **132**, 42482 (2015).
3. Ding, X., Kong, H., Qiao, M., Hu, Z. & Yu, M. Study on crystallization behaviors and properties of F-III fibers during hot drawing in supercritical carbon dioxide. *Polymers* **11**, 856 (2019).
4. Yang, C. *et al.*, Constructing mainstay-body structure in heterocyclic aramid fiber to simultaneously improve tensile strength and toughness. *Compos. B: Eng.* **202**, 108411 (2020).
5. Ran, S. *et al.*, Structural changes during deformation of Kevlar fibers via on-line synchrotron SAXS/WAXD techniques. *Polymer* **42**, 1601-1612 (2001).
6. Zhu, C. *et al.*, Relationship between performance and microvoids of aramid fibers revealed by two-dimensional small-angle X-ray scattering. *J. Appl. Crystallogr.* **46**, 1178-1186 (2013).
7. Xie, W. *et al.*, Dynamic strengthening of carbon nanotube fibers under extreme mechanical impulses. *Nano Lett.* **19**, 3519-3526 (2019).
8. OConnor, I., Hayden, H., Coleman, J. N. & Gunko, Y. K. High-strength, high-toughness composite fibers by swelling Kevlar in nanotube suspensions. *Small* **5**, 466-469 (2009).
9. Kumar, S. *et al.*, Synthesis, structure, and properties of PBO/SWNT composites. *Macromolecules* **35**, 9039-9043 (2002).
10. Zhou, C., Wang, S., Zhang, Y., Zhuang, Q. & Han, Z. In situ preparation and continuous fiber spinning of poly (*p*-phenylene benzobisoxazole) composites with oligo-hydroxyamide-functionalized multi-walled carbon nanotubes. *Polymer* **49**, 2520-2530 (2008).
11. Hu, Z. *et al.*, One-pot preparation and continuous spinning of carbon nanotube/poly (*p*-phenylene benzobisoxazole) copolymer fibers. *J. Mater. Chem.* **22**, 19863-19871 (2012).
12. Ruan, S., Gao, P. & Yu, T. X. Ultra-strong gel-spun UHMWPE fibers reinforced using multiwalled carbon nanotubes. *Polymer* **47**, 1604-1611 (2006).
13. Bencomo-Cisneros, J. A. *et al.*, Characterization of Kevlar-29 fibers by tensile tests and nanoindentation. *J. Alloys Compd.* **536**, S456-S459 (2012).
14. Li, J. *et al.*, Holey Reduced graphene oxide scaffolded heterocyclic aramid fibers with enhanced mechanical performance. *Adv. Funct. Mater.* 2200937 (2022).
15. Xiang, X. *et al.*, *In situ* twisting for stabilizing and toughening conductive graphene yarns. *Nanoscale* **9**, 11523-11529 (2017).
16. Xu, Z., Sun, H., Zhao, X., & Gao, C. Ultrastrong fibers assembled from giant graphene oxide sheets. *Adv. Mater.* **25**, 188-193 (2013).
17. Xu, Z. *et al.*, Ultrastiff and strong graphene fibers via full-scale synergetic defect engineering. *Adv. Mater.***28**, 6449-6456 (2016).
18. Xin, G. *et al.*, Microfluidics-enabled orientation and microstructure control of macroscopic graphene fibres. *Nat. Nanotechnol.* **14**, 168-175 (2019).
19. Li, P. *et al.*, Highly crystalline graphene fibers with superior strength and conductivities by plasticization spinning. *Adv. Funct. Mater.* **30**, 2006584 (2020).

20. Xin, G. *et al.*, Highly thermally conductive and mechanically strong graphene fibers. *Science* **349**, 1083-1087 (2015).

21. Taylor, L. W. *et al.*, Improved properties, increased production, and the path to broad adoption of carbon nanotube fibers. *Carbon* **171**, 689-694 (2021).

22. Zhang, X. *et al.*, Ultrastrong, stiff, and lightweight carbon-nanotube fibers. *Adv. Mater.* **19**, 4198-4201 (2007).

23. Ryu, S. *et al.*, Direct insulation-to-conduction transformation of adhesive catecholamine for simultaneous increases of electrical conductivity and mechanical strength of CNT fibers. *Adv. Mater.* **27**, 3250-3255 (2015).

24. Lee, D. *et al.*, Ultrahigh strength, modulus, and conductivity of graphitic fibers by macromolecular coalescence. *Sci. Adv.* **8**, eabn0939 (2022).

25. Kim, S. G. *et al.*, Ultrahigh strength and modulus of polyimide-carbon nanotube based carbon and graphitic fibers with superior electrical and thermal conductivities for advanced composite applications. *Compos. B: Eng.* **247**, 110342 (2022).

26. Liao, X. *et al.*, High strength in combination with high toughness in robust and sustainable polymeric materials. *Science* **366**, 1376-1379 (2019).

27. Ming, X. *et al.*, 2D-topology-seeded graphitization for highly thermally conductive carbon fibers. *Adv. Mater.* **34**, 2201867 (2022).

28. Gao, Z. *et al.*, Graphene reinforced carbon fibers. *Sci. Adv*. **6**, eaaz4191 (2020).

29. Eom, W. *et al.*, Microstructure-controlled polyacrylonitrile/graphene fibers over 1 gigapascal strength. *ACS Nano* **15**,13055-13064 (2021).

30. Kim. J. *et al.*, Longitudinal alignment effect of graphene oxide nanoribbon on properties of polyimide-based carbon fibers. *Carbon* **198**, 219-229 (2022).

31. Li, M. *et al.*, Robust carbon nanotube composite fibers: strong resistivities to protonation, oxidation, and ultrasonication. *Carbon* **146**, 627-635 (2019).

32. Kim, S. G. *et al.*, Ultrastrong hybrid fibers with tunable macromolecular interfaces of graphene oxide and carbon nanotube for multifunctional applications. *Adv. Sci.* **9**, 2203008 (2022).

33. Eom. W. *et al.*, Carbon nanotube-reduced graphene oxide fiber with high torsional strength from rheological hierarchy control. *Nat. Commun.* **12**, 396 (2021).

34. Yang, C. *et al.*, Constructing mainstay-body structure in heterocyclic aramid fiber to simultaneously improve tensile strength and toughness. *Compos. B: Eng.* **202**, 108411 (2020).

35. Ohta, Y. & Kajiwara, K. *Identification of Textile Fibers* (Woodhead Publishing, 2009).

36. Liu, Y., & Kumar, S. Recent progress in fabrication, structure, and properties of carbon fibers. *Polym. Rev.* **52**, 234-258 (2012).

37. Luo, L *et al.*, Analysis of structures and properties of representative heterocyclic aramid fibers in China and abroad. *China Synthetic Fiber Industry* **44**,91-95 (2021).
