# Supplementary material for: Fabricating strong and tough aramid fibers by small addition of carbon nanotubes
Source: Nat Commun. 2023 May 25;14:3019. doi: 10.1038/s41467-023-38701-4 (PMC10212957; doi:10.1038/s41467-023-38701-4)
Supplement: Supplementary file 3 — Description of Additional Supplementary Files [file 41467_2023_38701_MOESM3_ESM.pdf]

# Description of Additional Supplementary Files

**Supplementary Movie 1**: A series of sections derived from FIBSEM of HAFs

**Supplementary Movie 2**: A series of sections derived from FIBSEM of sa-SWNT-HAFs

**Supplementary Movie 3**: 3D observation of reconstructed void microstructure derived from FIBSEMT of HAFs

**Supplementary Movie 4**: 3D observation of reconstructed void microstructure derived from FIBSEMT of sa-SWNT-HAFs
